# Single-cell profiling of response to neoadjuvant chemo-immunotherapy in surgically resectable esophageal squamous cell carcinoma

**DOI:** 10.1186/s13073-024-01320-9

**Published:** 2024-04-02

**Authors:** Gang Ji, Qi Yang, Song Wang, Xiaolong Yan, Qiuxiang Ou, Li Gong, Jinbo Zhao, Yongan Zhou, Feng Tian, Jie Lei, Xiaorong Mu, Jian Wang, Tao Wang, Xiaoping Wang, Jianyong Sun, Jipeng Zhang, Chenghui Jia, Tao Jiang, Ming-gao Zhao, Qiang Lu

**Affiliations:** 1 Department of Digestive Surgery, Xijing Hospital, Air Force Medical University, No. 169 Changle West Road, Xi’an, 710032 China; 2 Precision Pharmacy & Drug Development Center, Department of Pharmacy, Tangdu Hospital, Air Force Medical University, No. 569 Xinsi Road, Xi’an, 710038 China; 3 Geneseeq Research Institute, Nanjing Geneseeq Technology Inc, Nanjing, 210000 Jiangsu China; 4 Department of Thoracic Surgery, Tangdu Hospital, Air Force Medical University, No. 569 Xinsi Road, Xi’an, 710038 China; 5 Department of Pathology, Tangdu Hospital, Air Force Medical University, No. 569 Xinsi Road, Xi’an, 710038 China; 6 Department of Thoracic Surgery, The First Affiliated Hospital of Xi’an Medical College, Xi’an, 710000 China

**Keywords:** Esophageal squamous cell carcinoma, Neoadjuvant therapy, Single-cell sequencing, Pathological response

## Abstract

**Background:**

The efficacy of neoadjuvant chemo-immunotherapy (NAT) in esophageal squamous cell carcinoma (ESCC) is challenged by the intricate interplay within the tumor microenvironment (TME). Unveiling the immune landscape of ESCC in the context of NAT could shed light on heterogeneity and optimize therapeutic strategies for patients.

**Methods:**

We analyzed single cells from 22 baseline and 24 post-NAT treatment samples of stage II/III ESCC patients to explore the association between the immune landscape and pathological response to neoadjuvant anti-PD-1 combination therapy, including pathological complete response (pCR), major pathological response (MPR), and incomplete pathological response (IPR).

**Results:**

Single-cell profiling identified 14 major cell subsets of cancer, immune, and stromal cells. Trajectory analysis unveiled an interesting link between cancer cell differentiation and pathological response to NAT. ESCC tumors enriched with less differentiated cancer cells exhibited a potentially favorable pathological response to NAT, while tumors enriched with clusters of more differentiated cancer cells may resist treatment. Deconvolution of transcriptomes in pre-treatment tumors identified gene signatures in response to NAT contributed by specific immune cell populations. Upregulated genes associated with better pathological responses in CD8 + effector T cells primarily involved interferon-gamma (IFNγ) signaling, neutrophil degranulation, and negative regulation of the T cell apoptotic process, whereas downregulated genes were dominated by those in the immune response-activating cell surface receptor signaling pathway. Natural killer cells in pre-treatment tumors from pCR patients showed a similar upregulation of gene expression in response to IFNγ but a downregulation of genes in the neutrophil-mediated immunity pathways. A decreased cellular contexture of regulatory T cells in ESCC TME indicated a potentially favorable pathological response to NAT. Cell–cell communication analysis revealed extensive interactions between CCL5 and its receptor CCR5 in various immune cells of baseline pCR tumors. Immune checkpoint interaction pairs, including CTLA4-CD86, TIGIT-PVR, LGALS9-HAVCR2, and TNFSF4-TNFRSF4, might serve as additional therapeutic targets for ICI therapy in ESCC.

**Conclusions:**

This pioneering study unveiled an intriguing association between cancer cell differentiation and pathological response in esophageal cancer patients, revealing distinct subgroups of tumors for which neoadjuvant chemo-immunotherapy might be effective. We also delineated the immune landscape of ESCC tumors in the context of clinical response to NAT, which provides clinical insights for better understanding how patients respond to the treatment and further identifying novel therapeutic targets for ESCC patients in the future.

**Supplementary Information:**

The online version contains supplementary material available at 10.1186/s13073-024-01320-9.

## Background

Esophageal cancer (EC) is the eighth most frequent cancer and the sixth leading cause of cancer-related mortality worldwide [1]. EC has two predominant histological subtypes, esophageal adenocarcinoma (EAC), and esophageal squamous cell carcinoma (ESCC), with distinct geographical and racial variabilities. Unlike Western nations, ESCC is particularly more prevalent in Central and Eastern China, constituting approximately 90% of all EC cases [2]. Despite incremental advances in diagnostics and therapeutics, ESCC has a dismal 5-year survival rate of 12–20% [3]. Neoadjuvant chemoradiotherapy followed by surgery has become the standard treatment for patients with resectable locally advanced ESCC [4]. However, nearly half of patients develop local recurrence or distant metastases following surgery, with an anticipated increase in toxicity levels and severe side effects [5, 6]. Therefore, there is an unmet clinical need to explore novel and effective treatments to improve patient survival.

In recent years, incorporating immune checkpoint inhibitors (ICIs) that target programmed cell death 1 (PD-1) with its ligand PD-L1 has emerged as a promising neoadjuvant treatment strategy in early-stage solid tumors, such as breast cancer, lung cancer, and ESCC [7–9]. Several clinical trials, including KEYNOTE 590 [10] and CheckMate 649 [11], have demonstrated promising antitumor activity and the safety of immunotherapies with or without chemotherapy in patients with advanced ESCC. Nevertheless, despite the encouraging preliminary results yielded by the use of ICIs in preoperative neoadjuvant therapy settings, several unsolved issues require attention. The identification of biomarkers that reflect intricate interactions between the tumor and the immune system will be pivotal in effectively stratifying patients who will truly benefit from ICI neoadjuvant therapy.

Positive PD-L1 expression has been reported in 18.9 to 45% of ESCC patients [12–15]. Although the relationship between PD-L1 expression and clinical outcomes in ESCC has been previously assessed, there is ongoing debate regarding whether PD-L1 expression is positively or negatively correlated with prognosis [16–18]. Theoretically, the extent of CD8 + T cell infiltration within the TME is associated with ICI response, given that blocking the PD-1/PD-L1 axis can rejuvenate the cytotoxic effect of CD8 + exhausted T cells [19]. However, recent studies harnessing neoadjuvant immunotherapy showed no significant difference in CD8 + T cells between responders and nonresponders [20–23]. Furthermore, prior studies have demonstrated that the upregulation of inflammatory and interferon-gamma (IFNγ) signaling-related gene signatures in inoperable EAC patients serve as on-treatment markers of ICI efficacy, while the elevated expression of E2F targets and genes related to extracellular matrix has been associated with tumor growth and resistance to ICI treatment [24]. The complex nature of IFNγ in the TME is continuously unfolding, and increasing evidence suggests that IFNγ may exert different functions depending on the immune context in which it is produced [25–29].

Single-cell RNA sequencing (scRNA-seq) has been arising as an unbiased, cutting-edge method that allows the grouping of cells based on their distinct transcriptional signatures without prior knowledge of genes and proteins of interest. Single-cell transcriptomic analysis offers an extraordinary opportunity to conduct an in-depth analysis of heterogeneous cells, including malignant cells, immune cells, and stromal cells. Indeed, much attention is currently focused on deconvolving inter-tumoral and intra-tumoral heterogeneity, which might imply various mechanisms to evade antitumor immune responses. Several recent studies have extensively investigated the intricate TME in esophageal cancer patients at the single-cell level in the context of neoadjuvant therapies; however, these studies have been constrained by relatively small sample sizes [24, 30, 31]. In this study, we undertake the scRNA-seq assay to uncover the key role of intra-tumoral cell type composition and their relationship to differential pathological responses to neoadjuvant therapy with PD-1 monoclonal antibodies plus chemotherapy in ESCC.

## Methods

### Participants and sample collection

Participants who had received immune therapy, such as monoclonal antibodies against programmed death-1 (PD-1)/PD-1 ligand (PD-L1) and cytotoxic T lymphocyte-associated antigen-4 (CTLA-4) or targeted therapies and radiotherapy were ineligible for this study. After screening, 22 patients who were histologically diagnosed with stage II to IV esophageal cancer between July 2020 and March 2021 and had not received the treatment mentioned above were included in this study. Relevant clinical information, such as sex, age, and treatment information, was retrieved from the database for each patient. The clinical stage was determined according to the American Joint Committee on Cancer (eighth edition) [32]. This study was conducted following the Declaration of Helsinki and was approved by the Institutional Ethics Committee of Tangdu Hospital, Fourth Military Medical University (No. 202102–23). Written informed consent was obtained from each patient before sample collection.

All 22 patients received neoadjuvant chemo-immunotherapy (NAT) comprising tislelizumab (BeiGene Co. Ltd, Shanghai, China) (*N* = 20) or camrelizumab (Jiangsu Hengrui Pharmaceuticals Co. Ltd) (*N* = 2), along with carboplatin/nedaplatin (Qilu Pharmaceutical Co. Ltd, Jinan, China) and albumin-bound paclitaxel (Qilu Pharmaceutical Co. Ltd, Jinan, China) for three cycles (3 weeks/cycle). Among these, 18 patients underwent curative-intent complete surgery following NAT.

**Fig.1:**
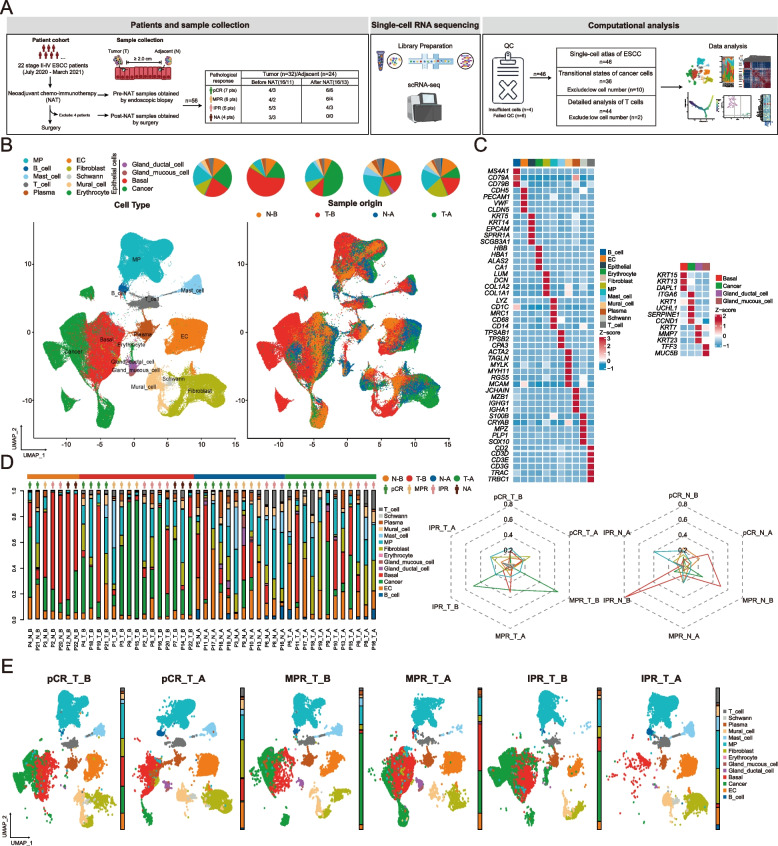
Single-cell atlas of ESCC. **A** Schematic demonstration of the experimental workflow for single-cell RNA sequencing and computational analysis. **B** UMAP embedding overlaid with unsupervised cluster cell type annotations (left), and sample origin annotations (right). Pie charts (top) demonstrate the proportion of each cell cluster based on the cell type or sample origin classifications. **C** Average expression profile of canonical marker genes to separate cell populations across 46 samples collected from 22 ESCC patients. **D** The proportion of cell subsets across 46 samples (left) and the proportion change of cells across 12 sample groups (right). **E** UMAP profiles delineating cell subsets collected from ESCC tumors, categorized based on treatment status and pathological response to neoadjuvant chemo-immunotherapy. pCR, pathological complete response; MPR, major pathological response; IPR, incomplete pathological response; T, tumor; N, normal tissue; B, pre-treatment; A, post-treatment

Tissue samples were obtained by endoscopic biopsy under general anesthetic during routine staging (pre-NAT T_B and N_B) or sampling of esophagectomy resection (post-NAT T_A and N_A) [30, 31]. Adjacent normal esophageal tissue biopsies (N_B) or tissue specimens (N_A) were obtained at least 2.0 cm distant from the matched tumor (Fig. 1A). Pathological response to NAT was evaluated based on the presence of viable residual tumor cells (VRTCs) in the resected primary tumor [33]. In particular, the presence of VRTCs ≥ 1% and ≤ 10% in the resected tumor and all resected lymph nodes was defined as the major pathological response (MPR), whereas the presence of ≤ 1% VRTCs in the resected specimen was defined as pathological complete response (pCR). The presence of > 10% VRTCs in the resected specimen was defined as an incomplete pathological response (IPR). Consequently, the study cohort was categorized into three groups: 7 cases of pCR, 6 cases of MPR, and 5 cases of IPR. This comprised a total of 32 tumor samples (16 T_B and 16 T_A, respectively) and 24 adjacent samples (11 N_B and 13 N_A, respectively) (Fig. 1A). After excluding poor-quality samples, there were 15 T_B samples, 7 N_B samples, 12 T_A samples, and 12 N_A samples subjected to scRNA-seq analysis (Additional file 1: Fig. S1A). Available samples were aggregated for uniform manifold projection (UMAP) dimensionality reduction to attain a holistic overview of the single-cell atlas of the ESCC TME. Principal findings regarding cancer and specific immune cell populations were exclusively based on pre-treatment tumor samples (T_B).

### Library preparation and single-cell RNA sequencing

Freshly excised samples were stored in the sCelLiVE® Tissue Preservation Solution (Singleron Biotechnology, Nanjing, China) on ice and transferred to the laboratory within 30 min after resection. All specimens were washed with Hanks Balanced Salt Solution (HBSS) three times, minced into 1-mm cubic pieces, and then digested with 3 mL sCelLiVE® Tissue Dissociation Solution (Singleron Biotechnology, Nanjing, China) at 37 °C for 15 min. The cell suspension was collected and filtered through a 40-μm sterile strainer. To remove red blood cells, the GEXSCOPE® red blood cell lysis buffer (RCLB) (Singleron Biotechnology, Nanjing, China) was added into the cell suspension at a ratio of 2:1 (RCLB: cell) and incubated at room temperature for 5 min. The mixture was then centrifuged at 300 × *g* for 5 min at 4℃. The supernatant was removed and the sediment was gently resuspended in phosphate-buffered saline (PBS) (HyClone, China).

scRNA-seq libraries were constructed as previously described [34]. In brief, single-cell suspensions (~ 2 × 10^5^ cells/mL) were loaded onto a microfluidic chip of the Singleron Matrix® Single Cell Processing System (Singleron Biotechnology, Nanjing, China). After the mRNA from each cell was labeled with a unique molecular identifier (UMI), single-cell suspensions were collected from the microfluidic chip, followed by reverse transcription and PCR amplification to obtain the scRNA-seq libraries. Individual libraries were diluted to 4 nM and paired-end sequenced on the NovaSeq 6000 sequencing system (Illumina, San Diego, USA).

### scRNA-seq data processing

The CeleScope (version 1.9.0, https://github.com/singleron-RD/CeleScope) pipeline was used to generate the gene expression matrix containing normalized gene counts versus cells per sample. The expression matrix was filtered for each sample dataset through the Seurat (v3.1.2) R toolkit [35]. The primary exclusion criteria are as follows: (1) cells with a gene count less than 200 or with a top 2% gene count; (2) cells with a maximum of 2% of UMI counts; (3) cells with mitochondrial content more than 50%; (4) genes expressed in less than five cells. The quality control data for scRNA-seq can be found in Additional file 2.

### Dimensionality reduction, clustering, and cell type annotation

The filtered raw count matrix was normalized by dividing by the total counts per cell and transformed by calculating the natural logarithm. The top 2000 variable genes were selected using the “FindVariableFeautres” function in Seurat (v3.1.2) [35]. Principal component analysis was then performed, and the top 20 principal components were subjected to dimensionality reduction and subsequent cell type clustering using the “FindClusters” function. Batch effect correction was performed using Harmony v1.0 [36]. Cell clustering was achieved by the Louvain algorithm, setting the resolution parameter to 0.8, and visualized by the Uniform Manifold Approximation and Projection (UMAP) method [37]. Cell-ID (v0.1.0, https://github.com/RausellLab/CelliD) [38] was used for automated annotation of cell types, followed by manual correction of cell types by classical markers in the SynEcoSys database (Singleron Biotechnology, Nanjing, China) [39–41]. All Seurat functions were run with default parameters unless otherwise specified.

### Inference of copy number variations from scRNA-seq data

Inferred copy number variations (CNVs) were analyzed using the inferCNV tool (v1.2.1; https://github.com/broadinstitute/inferCNV) [42] using non-malignant cells as baselines. In brief, genes expressed in more than three cells were sorted by their genomic locations on each chromosome. A sliding window comprising 101 genes was used to smooth the relative expression on each chromosome to remove the effect of gene-specific expression. The relative expression values were centered at 1, and the ceiling of the relative expression values was set to 1.5 standard deviations from the residual normalized expression levels. The R package Pheatmap (v1.0.12; https://github.com/raivokolde/pheatmap) [43] was used to visualize patients’ inferred CNV profiles.

### Single-cell trajectory analyses

SLICE (https://research.cchmc.org/pbge/slice.html) was applied to determine the single-cell entropy (scEntropy) of cancer cells [44]. The differentiation of cancer cells was subsequently delineated using the scEntropy-directed pseudotime trajectory generated by the Monocle 2 method (v2.22.0, https://www.bioconductor.org/packages/release/bioc/html/monocle.html) [45]. The “FindVariableFeatures” in Seurat (v3.1.2) was used to select highly variable genes from clusters. DDRTree, a reversed graph embedding algorithm in Monocle 2, was used to reduce dimension and reconstruct the temporal and bifurcation structure of the database based on global gene expression levels. The trajectory was visualized by the “plot_cell_trajectory” function in Monocle 2.

### Hotspot analysis

Hotspot (v0.9.1, https://github.com/YosefLab/Hotspot) was used to identify functional gene modules that illustrate heterogeneity within cancer cell subsets [46, 47]. Briefly, we used the “danb” model and selected the top 500 genes with the highest autocorrelation *z*-score for module identification. Modules were then identified using the “create_modules” function with the parameters: min_gene_threshold = 15 and fdr_threshold = 0.05. Module scores were calculated by using the “calculate_module_scores” function. Jaccard similarity coefficients were used to evaluate the transcriptional similarity between 12 cancer modules and signatures of cancer cell clusters.

### SCENIC analysis

Single-cell regulatory network inference and clustering (SCENIC), a computational method for network inference and motif discovery allowing high-confidence prediction of key regulators and their direct target genes, was performed using the R package “SCENIC” (v0.1.5; https://github.com/aertslab/SCENIC) [48, 49]. The co-expression modules were run by GRNBoost. We downloaded the motifs database for Homo sapiens from https://pyscenic.readthedocs.io/en/latest/. The input matrix consisted of the normalized expression values of the cells of interest.

### Differentially expressed gene analysis

Differentially expressed genes (DEGs) were identified by the “FindMarker” function in Seurat based on the Wilcoxon rank sum test. Genes expressed in more than 10% of cells in a cluster and with an average log (fold change) greater than 0.25 and an adjusted *P* value less than 0.05 were identified as DEGs. The cutoff for DEGs remains the same unless otherwise specified.

### Pathway enrichment analysis

Gene ontology (GO) and Kyoto Encyclopedia of Genes and Genomes (KEGG) analysis were performed using the “clusterProfiler” package in R [50]. GO gene sets include three categories, namely biological process (BP), cellular component (CC), and molecular function (MF). Pathways with an adjusted *P* value less than 0.05 were considered significantly enriched.

### Cell communication analysis

The CellPhoneDB (v2.1.0), equipped with a known ligand-receptor pair database, was used to predict cell–cell interaction [51, 52]. The number of ineffective distributions of reciprocal exchanges was set to 1000 and otherwise followed the default settings of the software. The predicted interaction pairs with a *P* value less than 0.05 and average log (fold change) greater than 0.1 were considered significant.

### Survival analysis

The Cancer Genome Atlas (TCGA)-esophageal carcinoma (ESCA) dataset, which includes bulk RNA-seq data and overall survival information for 159 patients, was obtained from cBioPortal (https://www.cbioportal.org/). To evaluate the relationship between the target gene set and patient prognosis, single sample gene set enrichment (ssGSVA) was used to calculate the score of each gene set for samples as previously described [53]. Samples were assigned to either high or low-expression groups using the best score as the cutoff. Kaplan–Meier curves were used to show differences in the overall survival of patients between high and low-expression groups for each gene set using the “Survival” package in R (https://github.com/therneau/survival).

### Statistical analysis

The unpaired two-sample Wilcoxon test was used to compare cell distribution within two groups of cells. An unpaired two-sample *t*-test was used to compare the mean gene expression or gene signatures within two groups of cells. The Jonckheere-Terpstra test was used to assess the statistical significance of the trends across IPR, MPR, and pCR groups. Log-rank test was used to compare the overall survival of subgroup patients. A two-sided *P* value of less than 0.05 was considered significant for all tests unless indicated otherwise (*P < 0.05, **P < 0.01, ***P < 0.001). All statistical analyses were performed in R (version 4.1.3).

## Results

### Patient overview

This study enrolled 22 stage II-IV ESCC patients, of whom 20 underwent neoadjuvant tislelizumab combined with chemotherapy, and two were treated with camrelizumab plus chemotherapy (Fig. 1A). The baseline clinical characteristics of patients are summarized in Table 1. In particular, 14 patients were male, and half were above 65 years of age. Only one patient was diagnosed with esophageal adenosquamous carcinoma (EASC). Among the 22 patients, 15 (68.2%) had PD-L1 TPS scores < 1%, 1 (4.5%) had TPS ranging from 1 to 50%, 4 (18.2%) had TPS scores ≥ 50%, and TPS scores of two patients were unknown.

**Table 1:** Clinical characteristics of patients (*N* = 22)

Characteristics	*N* (%)
Sex	
Female	8 (36.4)
Male	14 (63.6)
Age	
> 65	11 (50.0)
≤ 65	11 (50.0)
Pathological response	
IPR	5 (22.7)
MPR	6 (27.3)
pCR	7 (31.8)
Unknown	4 (18.2)
Smoking history	
Never smokers	14 (63.6)
Former/current smokers	7 (31.8)
Unknown	1 (4.55)
Clinical stage	
II	10 (45.5)
III	11 (50.0)
IV	1 (4.55)
Histological subtype	
ESCC	21 (95.5)
EASC	1 (4.55)
TPS	
< 1%	15 (68.2)
1% ≤ TPS < 50%	1 (4.5)
≥ 50%	4 (18.2)
Unknown	2 (9.1)
CPS	
< 1	10 (45.5)
1 ≤ TPS < 10	3 (13.6)
10 ≤ TPS < 50	3 (13.6)
≥ 50	4 (18.2)
Unknown	2 (9.1)

*IPR* incomplete pathological response, *MPR* major pathological response, *pCR* pathological complete response, *ESCC* esophageal squamous cell carcinoma, *EASC* esophageal adenosquamous carcinoma, *TPS* tumor proportion score, *CPS* combined positive score

Notably, four patients had an unknown pathological response status due to surgical cancellations. The rest of the 18 patients were categorized into three pathological response groups, including those with pathological complete response (pCR), major pathological response (MPR), and incomplete pathological response (IPR) (Fig. 1A; Additional file 1: Fig. S1A). The clinical characteristics of the remaining 18 patients with a definitive response to NAT are shown in Additional file 3: Table S1.

### Single-cell gene expression atlas of ESCC

To describe the transcriptional expression atlas of ESCC at the single-cell level, we performed scRNA-seq on all 46 samples, including tumor and normal adjacent tissues before and after NAT (Fig. 1A). By comparing the expression of canonical cell-type marker genes, we identified 14 distinct cell subsets totaling over 250,000 cells, including cancer, immune (including T, B, plasma, mononuclear phagocytes (MP), erythrocyte and mast cells), stromal (including endothelial cells (EC), mural cells and fibroblasts), parenchymal (including basal, gland mucous cells, and gland ductal cells), and Schwann cells (Fig. 1B–D; Additional file 1: Fig. S1). In post-treatment ESCC tumors, we observed a notable reduction in the proportion of cancer cells, accompanied by an augmentation in the presence of T cells (Fig. 1B). These results indicate a dynamic change within the ESCC TME in response to treatment. Meanwhile, UMAP embeddings of pre-treatment ESCC tumors, stratified by pathological response, displayed extensive overlap, implicating a profoundly intricate and heterogeneous TME among these patients (Fig. 1E).

### Plasticity of ESCC epithelial cells and their developmental trajectories into cancer cells

**Fig. 2:**
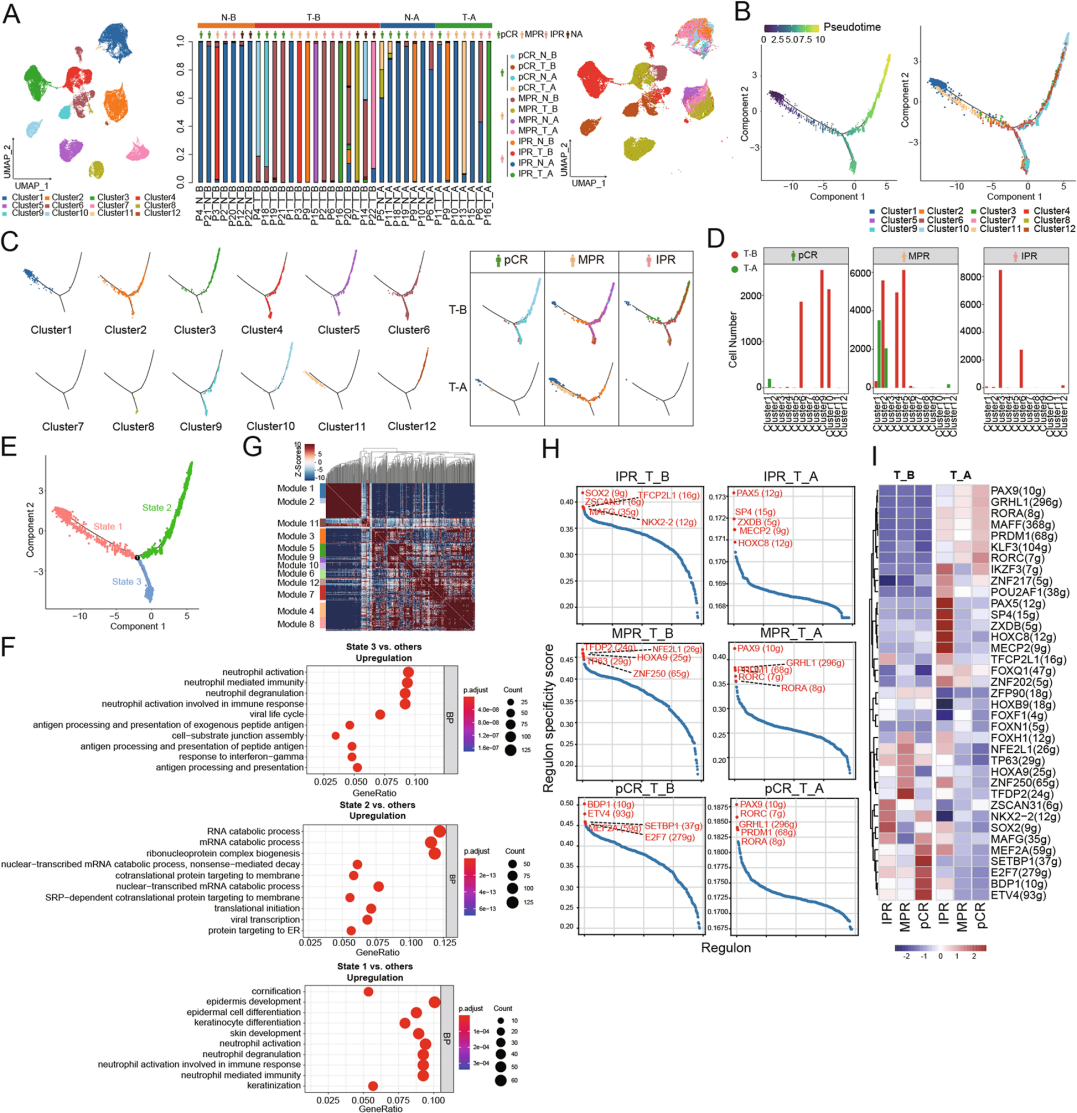
Transitional states of cancer cells revealed by trajectory mapping. **A** UMAP embedding of cancer cells overlaid with unsupervised cluster cell type annotations (left), proportional sample contributions to each cell type cluster (middle), and sample label (right). **B** Pseudotime trajectory of ESCC cancer cells in a two-dimensional state space inferred by the “Monocle 2” method. **C** Cancer cells mapped to the branched structure in the trajectories (left) and the distribution of cancer clusters in ESCC tumors stratified by treatment and pathological response (right). **D** Cell number count in cancer clusters identified in ESCC tumors of patients before (T_B) and after (T_A) neoadjuvant chemo-immunotherapy. **E** Pseudotime trajectory of ESCC cancer cells showing three differentiation states. **F** Investigation of biological processes (BP) through Gene Ontology (GO) pathway enrichment analysis in cancer cells across three differentiation states. **G** Heatmap shows the top 500 genes with significant autocorrelation grouped into 12 gene modules based on pairwise correlations of gene expression in cancer cells. **H** The scatter plots show the expression of the top five highly expressed regulons in each of the six cell subsets. **I** Heatmap of the area under the curve (AUC) scores of expression regulation by transcription factors in ESCC tumors stratified by treatment and pathological response, estimated by SCENIC

To analyze the developmental process and the expression heterogeneity of the malignant compartment, cancer cells were isolated based on the expression of canonical marker genes from ESCC tumors (Fig. 2A; Additional file 1: Fig. S2A). Through unsupervised clustering analysis, twelve distinct clusters of cancer cells were identified, and the extent of chromosomal copy number variations (CNVs) across the entire genome was inferred through a comprehensive analysis of scRNA-seq data and calculation of CNV scores [54, 55] (Additional file 1: Fig. S2B, C). Compared to non-malignant cells, all cancer cell clusters displayed heterogeneity in gene expression and CNV status. Notably, clusters 5 and 10 showed higher CNV scores compared to others, indicating a greater degree of chromosomal instability in their genomic profiles.

The dynamic characteristics and heterogeneity of malignant cancer cells were further investigated by trajectory analysis. To determine the start point of the inferred pseudotemporal cancer cell ordering, we first applied SLICE (*S*ingle Cell *L*ineage *I*nference Using *C*ell Expression Similarity and *E*ntropy) to quantify single-cell entropy (scEntropy) of each cancer cell type [44]. As entropy inversely correlates with cell differentiation state, cells with lower scEntropy had more well-defined cell fates and functionalities compared to those clusters with higher scEntropy. In this context, clusters 1 and 11 exhibited a significantly lower entropy compared to others, marking the start point in trajectory mapping (Additional file 1: Fig. S2D). Subsequently, we used the “Monocle 2” method [56–60] to construct entropy-directed transitional trajectories of cancer cells (Fig. 2B). Notably, differentiation trajectory analysis revealed a branched structure of cancer cells, with several clusters of cancer cells positioned along the extended branch away from the start point, suggesting that these cell clusters were likely associated with higher functional uncertainty and greater differentiation potential (Fig. 2C). Interestingly, we observed that less differentiated clusters (higher entropy, such as clusters 2, 4, 5, 9, 10) were primarily identified in pre-treatment tumor samples of pCR and MPR patients, while more differentiated clusters (lower entropy, such as clusters 1 and 11) were predominantly found in post-treatment tumor samples (Fig. 2D). These observations suggest an interesting link between cancer cell differentiation and the pathological response to neoadjuvant chemo-immunotherapy. Specifically, ESCC tumors enriched with less differentiated cancer cells might be associated with a better pathological response to neoadjuvant chemo-immunotherapy, whereas tumors enriched with clusters of more differentiated cancer cells may resist the treatment. Likewise, the pseudotime faceted map of paired tumors categorized by response displayed a consistent pattern, with a better pathological response observed in tumors enriched with cancer clusters characterized by higher entropies (Additional file 1: Fig. S2E). Nevertheless, it is crucial to recognize that scRNA-seq inherently presents limitations in precisely calculating cell proportions, as demonstrated by the underrepresentation of cancer cells in post-treatment tumor samples from IPR patients. Consequently, these results should be approached with caution, and future validation is imperative.

Furthermore, we analyzed the transcriptomic changes of cancer cells associated with transitional states (Fig. 2E, F). Three phases of differentiation were identified. Cancer cells in state 3, which positively responded to NAT, exhibited significantly elevated expression levels of immune-related genes, including those implicated in neutrophil activation, antigen processing and presentation, and response to IFNγ (Fig. 2F). Conversely, cancer cells mapped to state 2 showed significantly increased expression of genes associated with mRNA catabolic processes. On the other hand, state 1 was predominantly occupied by cancer cells characterized by higher expression of genes involved in epidermal development. These results were consistent with the dynamic changes in gene expression of cancer cells during the transition (Additional file 1: Fig. S2F, G). Collectively, these findings suggest that state 3 likely encompassed cancer cells actively engaged in immune responses, whereas state 1 appeared to consist primarily of epithelial-like cells at the transient stage.

### Dissecting the transcriptomic inter-tumor heterogeneity of cancer cells in ESCC

Hotspot analysis [46] was performed to explore informative gene modules characterized by distinct expression patterns and their correlation with pathological response to NAT. It is noteworthy that unsupervised clustering of cancer cells, as a data-driven method, facilitates the identification of subpopulations of cancer cells sharing similar expression patterns. On the other hand, hotspot analysis represents a hypothesis-driven approach, wherein specific gene sets and pathways are predefined based on prior knowledge or biological significance. Here, we identified 12 distinct gene modules corresponding to the 12 previously annotated cancer cell clusters in ESCC (Fig. 2G; Additional file 1: Fig. S2H). Cancer cluster 3 was predominantly observed in pre-treatment tumor samples of IPR patients and exhibited a strong correlation with module 3 (Fig. 2D; Additional file 1: Fig. S2H). However, it remains inconclusive whether the significant reduction of cluster 3 in post-treatment samples is correlated with the efficacy of NAT due to the limited representation of cancer cells in IPR_T_A samples (Fig. 1E). On the other hand, cancer cluster 1 with lower entropies was significantly correlated with module 1 (Jaccard similarity coefficient = 0.4). Consistent with the notion that cluster 1 cells displayed a stronger resemblance to normal epithelial cells, Gene Ontology (GO) enrichment analysis revealed that module 1 was dominated by genes associated with epidermal cell differentiation and development (Additional file 1: Fig. S2I). Notable genes within this category included *EMP1*, *SCEL*, *SPINK5*, *ANXA1*, *SPRR3*, *PPL*, *CNFN*, and *TGM3*. In contrast, gene modules associated with pathological complete response to NAT were primarily implicated in biological processes such as extracellular matrix organization (module 6), and activation of protein complex assembly (module 9) (Additional file 1: Fig. S2J). Regarding gene modules significantly contributing to MPR, the results of GO functional analysis unveiled a noteworthy enrichment of genes associated with epidermis development and response to type I interferon (module 7), response to hypoxia (module 10), and protein targeting to ER (module 4) (Additional file 1: Fig. S2K).

Given the unfortunate unavailability of survival data from the study cohort, we conducted an exploratory analysis utilizing the TCGA-ESCA dataset (*N* = 159). A Cox regression model was applied to investigate the relationship between cancer-associated gene modules and the patient’s overall survival (OS). Remarkably, higher expression of gene modules 6 and 12 was significantly associated with a more favorable prognosis of patients (*P* = 0.039 and 0.044, respectively; Additional file 1: Fig. S2L, M). In contrast, cluster 10-associated module 9 and cluster 5-associated module 10 were likely associated with a worse prognosis, though the difference did not reach statistical significance (Additional file 1: Fig. S2H, L). Nevertheless, it is worth noting that bulk RNA-seq data was used rather than scRNA-seq data and that the external dataset adheres to a different treatment regimen from the clinical settings in our analysis. Further exploration and validation of the association between cancer cell differentiation and immunotherapy-related patient prognosis are warranted in future studies.

We also employed SCENIC [49] to identify specific combinations of transcription factors that govern the expression of their respective target genes within cancer cells at the single-cell level. The top 5 regulons in cancer cells from patients exhibiting various responses to neoadjuvant anti-PD-1 combination therapy showed substantial differences in their expression patterns (Fig. 2H). Notably, pre-treatment pCR tumors (pCR_T_B) showed an upward trend in the expression of *ZNF217* and *MEF2A*, whereas a downward trend was observed in the expression of *TFCP2L1*, *ZSCAN31*, and *SOX2* compared to the other two groups (all *P* < 0.001, Jonckheere-Terpstra test) (Fig. 2I). Importantly, SOX2 has been extensively studied in the context of cancer, playing a pivotal role in tumor cell proliferation, cancer metastasis, and response to therapies [61]. Accumulating evidence suggests that SOX2 promotes cancer cell stemness, and its overexpression is associated with lymph node metastasis and poor prognosis in cancer patients [62]. Collectively, these findings suggest that the dynamic change in these transcription factors and their associated regulatory networks might be associated with the pathological response of patients who received NAT.

While PD-L1 expression has been employed to predict the efficacy of pembrolizumab in advanced EC [63], the predictive role of PD-L1 expression remains inconclusive for neoadjuvant therapy in EC patients. In our study, a total of 14 patients exhibited TPS scores < 50% or CPS scores < 50, thereby being classified as having PD-L1 low expression. Among these patients, 9 individuals (64.3%) exhibited pCR or MPR to NAT and five demonstrated IPR examined by conventional histologic approach (Additional file 3: Table S1). Dissecting the transcriptomes of cancer cells at single-cell resolution in low PD-L1 patients may provide significant clinical insights for understanding how these patients respond to neoadjuvant chemo-immunotherapy. In patients exhibiting low PD-L1 expression, a notable upregulation of genes associated with the protein targeting pathway was observed in tumors that achieved pCR. Conversely, in IPR tumors, there was an increased expression of genes associated with responding to oxidative stress (Additional file 1: Fig. S2N-P). These findings collectively suggest a plausible connection between the activation of these transcriptional signatures in cancer cells and the different pathological responses to NAT observed in patients with low PD-L1 expression.

### Increased immune surveillance associated with better pathological response revealed by ESCC TME

**Fig. 3:**
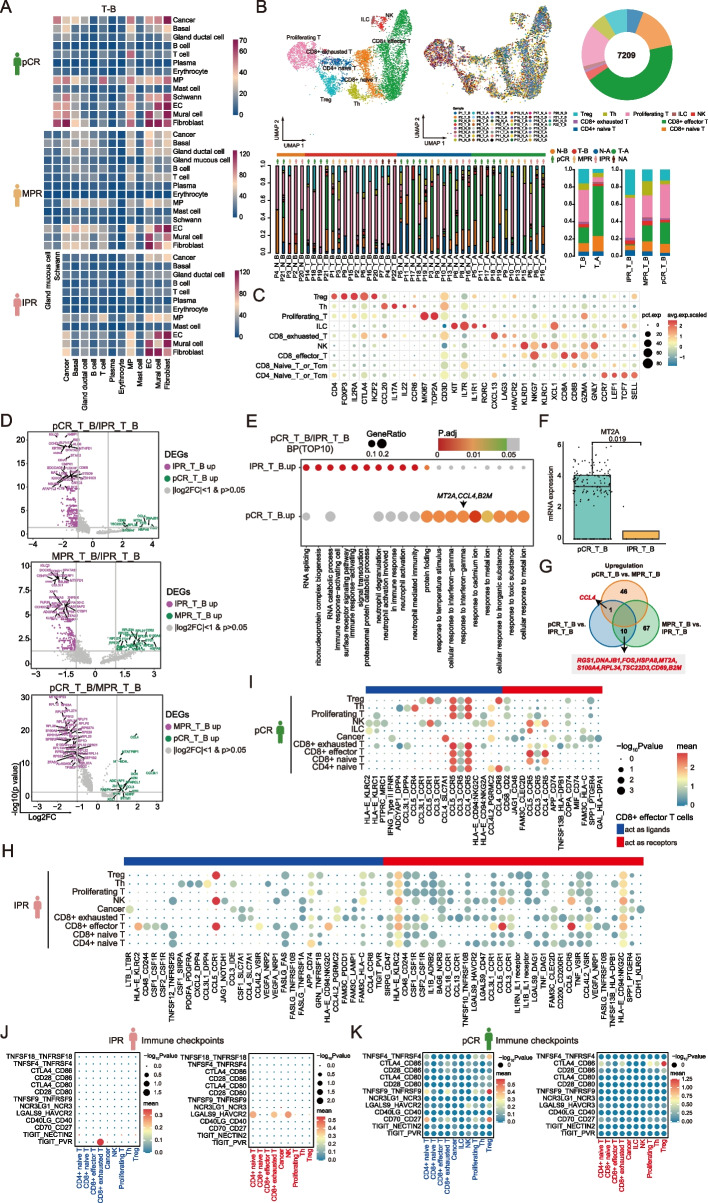
Characterization of CD8 + effector T cells within the ESCC TME. **A** The CellPhoneDB-generated heatmap shows the count of cell–cell interactions in baseline tumors obtained from pCR, MPR, and IPR patients. **B** UMAP embedding of T cells overlaid with cluster cell type annotations (left), sample label (middle), and proportional sample contributions to each cell type cluster (right). **C** Dot plots of canonical T cell marker gene expression in each T cell lineage. Dot size and color indicate the fraction of expressing cells and normalized expression levels, respectively. **D** Volcano plots show the differentially expressed genes (DEGs) in subgroup analysis. **E** Gene Ontology (GO) pathway enrichment analysis of the top 10 enriched biological processes (BP) for baseline ESCC tumor in pCR patients compared to IPR patients. **F** Box plots show the mRNA expression of *MT2A* in baseline ESCC tumors of pCR and IPR patients at the single-cell level. **G** The Venn diagram shows the intersections of three gene sets. Gene sets were based on DEG analysis comparing baseline tumors of patients with different pathological responses to neoadjuvant chemo-immunotherapy. **H, I** Dot plots show the top 30 (based on the expression level) ligand-receptor interactions from CD8 + effector T cells obtained from baseline ESCC tumors of IPR (**H**) and pCR patients (**I**). The size of the circle represents the *P* values, and the color of the circle indicates the average expression level of interacting pairs. The cell clusters labeled in blue and red on the *x*-axis indicate that CD8 + effector T cells act as ligands and receptors in the interaction pairs, respectively. **J, K** Dot plots illustrate a range of immune checkpoint interaction pairs involving CD8 + effector T cells and various other cell types obtained from pre-treatment ESCC tumors of IPR (**J**) and pCR patients (**K**)

We analyzed cell–cell communication across all cell types within the ESCC TME using the “CellphoneDB” method. The results revealed a positive correlation between TME activity and the patient’s pathological response to NAT, with a notably higher number of ligand-receptor pairs (LRPs) observed in baseline pCR tumors (Fig. 3A). We then categorized T cells based on canonical marker gene expression, identifying nine subsets totaling 7209 cells (Fig. 3B, C). Particularly noteworthy was the significantly greater proportion of CD8 + effector T cells in baseline pCR tumors compared to IPR tumors (Fig. 3B). Meanwhile, T cells exhibited stronger interactions with both MP cells and within their own population (Additional file 1: Fig. S3A, B). These findings suggest a positive association between CD8 + effector T cell infiltration and improved pathological response to NAT in ESCC.

### Elevated expression of IFNγ-responsive gene signature in CD8 + effector T cells predicts pathological response of ESCC patients to NAT

Transcriptional analysis was performed to identify treatment response-related molecular signatures in CD8 + effector T cells at the single-cell level. Emphasis was placed on identifying DEGs and enriched pathways within pre-treatment ESCC tumors of pCR and IPR patients who exhibit the most pronounced variability in response to NAT (Fig. 3D). The expression levels of genes involved in the cellular response to IFNγ (such as *MT2A*, *CCL4*, and *B2M*) were notably upregulated in baseline pCR tumors compared to those in IPR tumors (Fig. 3E, F). Of note, a decreased expression of metallothionein (MT) family genes, including *MT2A*, *MT1X*, and *MT1E*, has been previously associated with resistance to anti-PD-1 therapy in ESCC [64]. While *MT2A* expression was significantly higher in MPR patients compared to those with IPR (*P* = 0.008), the difference did not reach statistical significance (Additional file 1: Fig. S3C-F). These findings suggest that the functional significance of CD8 + effector T cells in baseline MPR patients may resemble that in pCR patients rather than IPR patients. Assessing DEGs across various comparison groups, we found that upregulated genes linked to better pathological responses were mainly markers associated with cellular response to IFNγ (*CCL4*, *MT2A*, and *B2M*), neutrophil degranulation (*B2M* and *HSPA8*), regulation of I-kappaB kinase/NF-kappaB signaling (*S100A4*), and negative regulation of T cell apoptotic process (*TSC22D3*) (Fig. 3G). In contrast, downregulated gene signatures in baseline ESCC tumors of patients with better NAT-associated pathological response were primarily involved in the immune response-activating cell surface receptor signaling pathway (such as *IGHG4*, *IGHA1*, *C3AR1*, *FCER1G*, *TYROBP*, *AAPL1*, and *LPXN*) (Fig. 3E; Additional file 1: Fig. S3G). Among these genes, higher expression of *C3AR1*, *FCER1G*, and *AAPL1* has been previously linked to an unfavorable prognosis in ESCC patients [65, 66].

To gain further insight into potential treatment strategies, we examined the top 30 ligand-receptor interactions between CD8 + effector T cells and cancer cells, as well as other T cell subsets. We observed a significantly lower number of cell–cell interactions in baseline tumors of IPR patients compared to those with pCR (Fig. 3H, I). In both IPR and pCR tumors, CCL5 was commonly expressed as a ligand on CD8 + effector T cells, with its receptor CCR1 expressed on NK cells and Treg cells. However, the identification of CCL5-CCR1 interactions within CD8 + effector T cells was observed only in baseline ESCC tumors of pCR patients. In contrast, CCL5-CCR5 interactions were widely expressed across various cell types in pCR tumors at baseline, except for cancer cells, NK cells, and innate lymphoid cells (ILC). Besides, CCL3 and CCL4 may also stimulate cell–cell communications through most immune cell subsets by binding to its receptor CCR5. Through assessment of the diverse expression patterns of immune checkpoints, we noticed a significant enrichment of interactions, including TIGIT-PVR and LGALS9-HAVCR2 in baseline IPR tumors, suggesting novel immunotherapy targets for ESCC patients who may not benefit from NAT (Fig. 3J, K).

Next, transcriptional changes in CD8 + effector T cells associated with pathological response to NAT were examined using pCR and IPR patients with low PD-L1 expression. Consistently, pCR patients with low PD-L1 expression exhibited higher expression levels of genes involved in the cellular response to IFNγ, along with lower expression of genes associated with immune response-activating signaling transduction pathway (Additional file 1: Fig. S3H-J). Overall, the recapitulation of GO enrichment analysis in low PD-L1 patients suggested that increased expression of IFNγ-responsive gene signatures and decreased expression of genes related to immune response-activating signaling pathway in CD8 + effector T cells might predict a favorable pathological response in locally advanced ESCC patients who underwent NAT.

### NK cells constitute a minor population but induce a cytotoxic cellular response in the ESCC TME

**Fig. 4:**
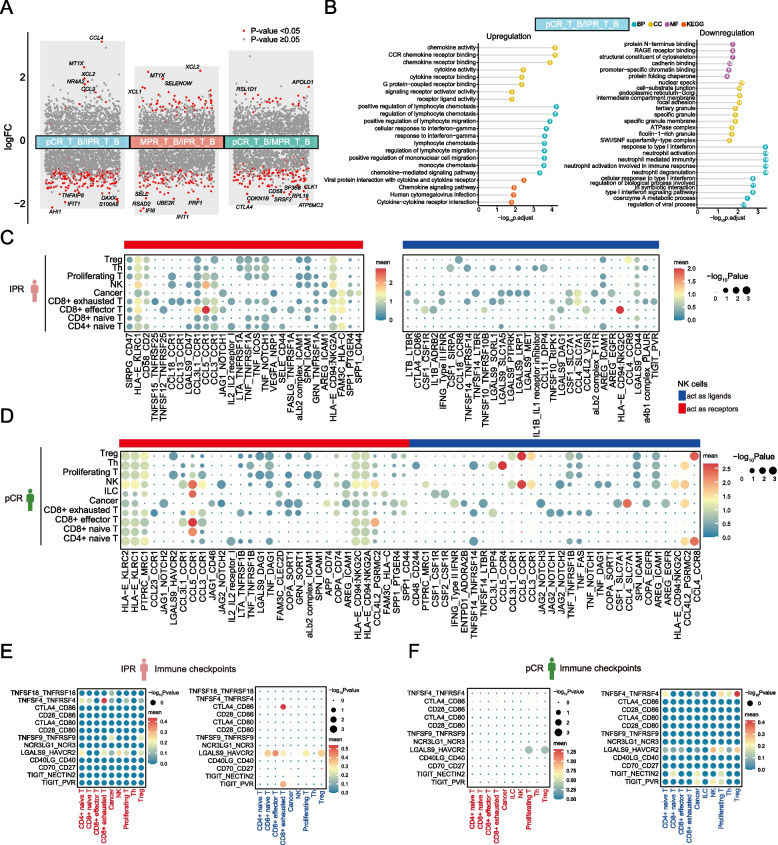
Transcriptional analysis of NK cells in baseline tumors of pCR and IPR patients. **A** Differentially expressed genes (DEGs) in subgroup analysis. Significant *P* values were labeled in red, and the *y*-axis represents the log2 fold change. **B** GO and KEGG pathway enrichment analysis for baseline ESCC tumor in pCR patients compared to IPR patients. The length and color of the bars represent enrichment significance and classifications. The number inside the spheres represents the number of related mRNAs enriched in the specific pathway. **C, D** Dot plots show the top 30 ligand-receptor interactions from NK cells obtained from baseline ESCC tumors of IPR (**C**) and pCR patients (**D**). The size of the circle represents the *P* values, and the color of the circle indicates the average expression level of interacting pairs. The cell clusters labeled in blue and red on the *x*-axis indicate that NK cells act as ligands and receptors in the interaction pairs, respectively. **E, F** Dot plots show the immune checkpoint ligand-receptor interactions from NK cells obtained from baseline ESCC tumors of IPR (**E**) and pCR patients (**F**)

NK cells constitute a unique subset of lymphocytes larger than T cells and B cells, capable of rapidly targeting and eliminating specific cells without prior immunization or MHC restriction [67, 68]. Our study identified a lower proportion of NK cells in pCR patients compared to those in non-pCR patients following NAT (Fig. 3B). Similarly, we assessed DEGs in the subgroup analysis (Fig. 4A, B; Additional file 1: Fig. S4A-C). Pathway enrichment analysis revealed that genes significantly upregulated in patients exhibiting a positive pathological response to NAT were primarily involved in enhancing lymphocyte chemotaxis (such as *CCL4*, *CCL3*, and *XCL2*) and regulating T cell-mediated immunity (*B2M* and *KLRD1*) (Additional file 1: Fig. S4A). Compared to IPR patients, those achieving pCR showed downregulation in genes associated with neutrophil-mediated immunity pathways, including *CXCL1* and *ITGB2* (Fig. 4B).

We then investigated the interactions between NK cells and various cell types. The expression of LRPs involving NK cells and immune cells, such as CCL5-CCR4, CCL5-CCR1, and CCL4-CCR8, were exclusively observed in pCR patients before NAT but not in IPR patients (Fig. 4C, D). Furthermore, while the CCL5-CCR1 interaction was detected between CD8 + effector T cells and NK cells in both pre-treatment pCR patients and IPR patients, this interaction pair was also identified in pCR patients between CD8 + naïve T cells and NK cells, as well as within the NK cell population. A range of immune checkpoint pairs, such as TNFSF4-TNFRSF4, CTLA4-CD86, LGALS9-HAVCR2, and TIGIT-PVR, between NK cells and CD8 + effector/exhausted T cells, were significantly enriched in pre-treatment IPR patients, indicating additional potential targets for immunotherapy in nonresponders following the current treatment regimen (Fig. 4E, F).

Similar to what was found in CD8 + effector T cells, in low PD-L1 expression pCR patients, NK cells exhibited a markedly elevated expression of genes involved in the cellular response to IFNγ, as well as those implicated in the response to interleukin-1, such as *CCL4*, *CCL3*, *CCL5*, *XCL1*, *UBB*, *ANXA1*, and *XCL2* (Additional file 1: Fig. S4D). Conversely, in IPR patients with low PD-L1 expression, there was a significant upregulation of genes associated with antiviral responses and those pivotal in RNA catabolic processes.

### Decreased proportions of Treg cells in ESCC indicate better pathological response following NAT

**Fig. 5:**
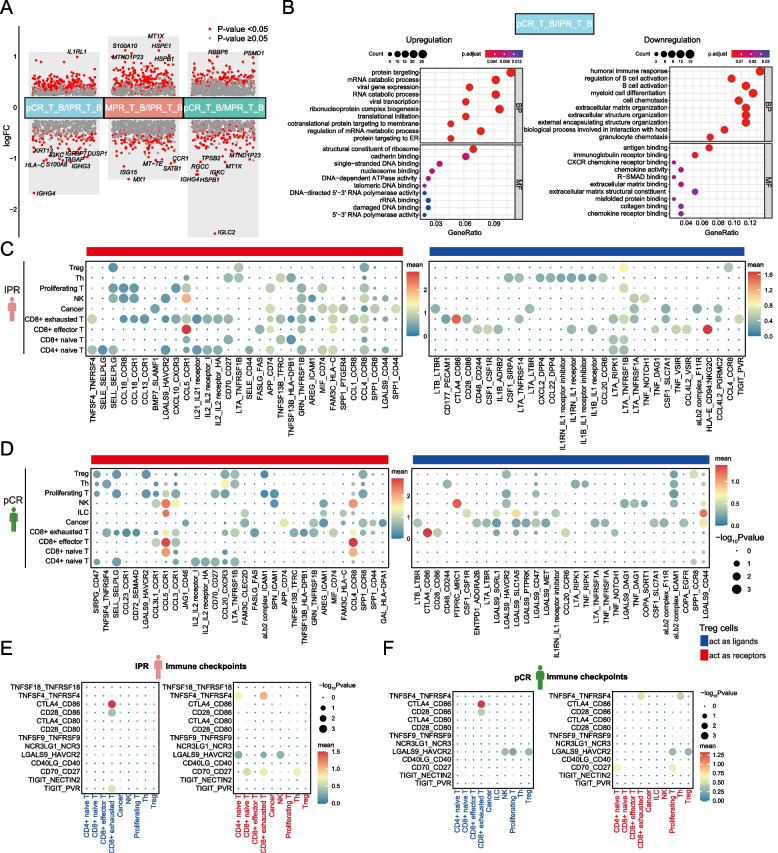
Regulatory T cells play an immunosuppressive role in TME. **A** Differentially expressed genes (DEGs) in subgroup analysis. Significant *P* values were labeled in red, and the *y*-axis represents the log2 fold change. The top 10 DEGs were denoted. **B** GO pathway enrichment analysis for baseline ESCC tumor in pCR patients compared to IPR patients. The *x*-axis represents the ratio of mRNAs enriched in GO terms. The *y*-axis represents the enriched pathway. The color and size of each bubble represent enrichment significance and the number of related mRNAs enriched in the pathway, respectively. **C, D** Dot plots illustrate the top 30 ligand-receptor interactions from Treg cells obtained from baseline ESCC tumors of IPR (**C**) and pCR patients (**D**). The size of the circle represents the *P* values, and the color of the circle indicates the average expression level of interacting pairs. The cell clusters labeled in blue and red on the *x*-axis indicate that Treg cells act as ligands and receptors in the interaction pairs, respectively. **E, F** Dot plots show the immune checkpoint ligand-receptor interactions from Treg cells obtained from baseline ESCC tumors of IPR (**E**) and pCR patients (**F**)

Treg cells, originating in the thymus, exert regulatory controls over various immune cells including T cells, B cells, NK cells, dendritic cells (DCs), and macrophages through both humoral and cell–cell contact mechanisms [69]. The infiltration of Treg cells is linked to poor prognosis due to their fundamental suppression mechanisms involving CTLA4, IL-2, IL-10, and other immune-suppressive cytokines and substances [69, 70]. In this study, we observed a decline in Treg cell numbers among ESCC patients exhibiting better pathological responses to NAT, consistent with their suppressive role within the TME (Fig. 3B). Then, we performed DEG analysis between patient subgroups categorized based on their pathological response to NAT (Fig. 5A, B; Additional file 1: Fig. S5). Comparison of baseline tumor samples from the two patient cohorts with the most variable responses to NAT revealed a significant downregulation of genes in pCR patients involved the humoral immune response (such as *S100A8*, *IGHG4*, *S100A9*, *IGKC*, *IGLC3*, *IGHG3*, *RGCC*, *HLA-A*, *IGHG1*, *CXCL8*, *CXCL1*, *A2M*, *KRT6A*, *IGHA1*, and *CXCL2*), B cell activation (such as *IGHG4*, *IGKC*, *IGLC3*, *IGHG3*, *IGHG1*, *BATF*, *TNFRSF4*, *RASGRP1*, *IGHA1*, *NDFIP1*, *ZFP36L1*, *TBC1D10C*, and*CTLA4*), and myeloid cell differentiation (such as *JUN*, *HSPA1B*, *HSPA1A*, *JUNB*, *ZFP36*, *ADAR*, *PIP4K2A*, *NFKBIA*, *NR4A3*, *FOS*, and *GNAS*) (Fig. 5B). As protein targeting, ribonucleoprotein complex biogenesis, and mRNA catabolic process were identified as the top three signaling pathways significantly enriched in pCR patients, we reasoned that Treg cells may contribute to improved clinical responses to NAT by maintaining cellular homeostasis, fostering self-tolerance, and facilitating tissue repair.

The examination of cell–cell interactions revealed that Treg cells displayed restricted interactions with other T cell subsets in IPR patients, whereas the communication was slightly intensified in pCR patients by the presence of CCL5-CCR1 and CCL4-CCR8 interactions (Fig. 5C, D). Immune checkpoint pairs, such as TIGIT-PVR, TNFSF4-TNFRSF4, LGALS9-HAVCR2, and CD70-CD27, were identified in baseline IPR patients as potential targets for immunotherapy (Fig. 5E, F).

## Discussion

Recent studies have undertaken scRNA-seq analyses to uncover the mechanisms driving the response of esophageal cancer to neoadjuvant chemotherapy with or without concurrent radiotherapy contributed by immune cells in the TME [30, 31]. Nevertheless, it is crucial to emphasize that nearly half of patients still encounter local recurrence or distant metastases after preoperative neoadjuvant chemoradiotherapy followed by surgery [20]. Hence, exploring novel and effective treatments remains an urgent need for improved survival of EC patients. Our study employed scRNA-seq to evaluate immune contexture and their correlation with pathological response to the emerging neoadjuvant chemo-immunotherapy, which holds significant clinical implications for the precise treatment of resectable ESCC patients.

The present investigation has yielded several significant findings. Firstly, we have deciphered the complicated compositions of the TME in ESCC using scRNA-seq, which identified 14 major cell clusters, including cancer, immune, stromal, and Schwann cells. At baseline, the TME was predominantly composed of epithelial cells, particularly cancer cells, followed by MP cells. Neoadjuvant chemo-immunotherapy may modulate cell type contexture in several ways. Most notable was a reduction in the proportion of cancer cells and an increase in T cells following therapy, indicating a dynamic change within the ESCC TME in response to treatment. Previous research has shown that neoadjuvant chemotherapy, whether administered alone or in combination with neoadjuvant radiotherapy, resulted in an augmentation of CD8 + T effector cell infiltration and a reduction in Treg cell populations in esophageal cancer patients [30, 31]. Consistently, we observed similar modulations of these T cell repertoire stratified before or after NAT, suggesting a potential synergy of different types of preoperative neoadjuvant therapies. Furthermore, patients across different response groups showed both intra- and inter-tumoral heterogeneity in their pre-treatment tumor samples, emphasizing the need for a more comprehensive investigation of the heterogeneous responses of malignant and immune cell components to NAT. Nonetheless, it is important to acknowledge that scRNA-seq has inherent limitations in accurately determining cell proportions. Therefore, conclusions drawn regarding proportions should be approached with caution. As evidence, we observed an underestimated proportion of cancer cells in post-treatment tumor samples from IPR patients.

Secondly, we discovered a potential link between tumor cell differentiation states and various major expression programs, which may potentially impact the pathological response to neoadjuvant chemo-immunotherapy. Worth noting that single-cell trajectories were constructed using the “Monocle 2” algorithm to elucidate the diverse cell fates of cancer cells within ESCC tumors. Our study did not propose any direct evolutionary/developmental relationships among individual tumors that are inherently genetically distinct. The results showed that the more differentiated cancer cells located at the pre-branch (state 1) may exhibit a closer resemblance to normal epithelial cells. On the contrary, less differentiated clusters (higher tumor stemness) positioned at the terminal point in the trajectory may display a stronger likeness to stem cells, characterized by a higher level of functional uncertainty. These cells were likely associated with neutrophil-mediated immunity and the processing and presentation of antigens. Notably, they are more prone to exhibiting a favorable pathological response to NAT. In particular, we observed a remarkable decrease in the number of cells within post-NAT tumors in cancer clusters 2, 4, 5, 9, and 10, whereas clusters 1 and 11 may potentially represent cell subsets demonstrating an adaptive immune resistance to NAT.

Thirdly, we utilized specific immune cell populations and performed a comprehensive analysis of DEGs and pathways among subgroup patients manifesting different pathological responses to NAT, with particular emphasis on pCR and IPR patients who demonstrate the most divergent clinical responses to therapy. Notably, Carroll et al. have reported an ICI-responsive gene signature known as INCITE [24]. The upregulation of INCITE genes involved in various inflammatory and IFNγ-related pathways could induce tumor shrinkage in EAC patients. Consistent with this finding, we noted a significant upregulation of genes associated with cellular response to IFNγ in both CD8 + effector T cells and NK cells of baseline pCR tumors compared to that in IPR tumors. Among these DEGs, *MT2A* may serve as a promising favorable prognostic indicator in ESCC patients undergoing neoadjuvant anti-PD-1 combined therapy, whereas *C3AR1* [65], *FCER1G*, *APPL1* [66], *S100A8* [71], *S100A9*, *CXCL1* [72], and *ITGB2* [73] might be associated with an unfavorable prognosis in ESCC. The finding that IFNγ-responsive genes correlate with better pathological response to NAT suggests that the immune response plays a crucial role in determining treatment efficacy. The potential of IFNγ to stimulate lymphocyte and macrophage functions highlights its significance as a therapeutic target in treating ESCC patients.

Furthermore, T cells express a broad spectrum of ligands and receptors for chemokines, cytokines, checkpoint molecules, and growth factors. The aberrant expression of the C–C chemokine ligand 5/C–C chemokine receptor type 5 (CCL5-CCR5) axis has been recognized as a pivotal contributor to tumor progression, facilitating a more conducive TME for hematological malignancies and various solid tumors [74, 75]. However, CCL5 may also augment the immunotherapy response by recruiting antitumor T cells and dendritic cells to the TME [76–79]. Here, we demonstrated extensive interaction between CCL5 and its specific receptor CCR5 in various immune cells of pre-treatment pCR patients. In contrast, very low levels or absence of the CCL5-CCR5 interaction pair could be detected in baseline tumor samples of IPR patients. These results suggest that the binding of CCL5 to its specific receptor CCR5 may facilitate cooperation among diverse immune cell types, stimulating the cytotoxic function of CD8 + effector T cells. It is also worth mentioning that CCR5 is a promiscuous G protein-coupled receptor that binds with high affinity to not only CCL5 but also CCL3 and CCL4 [80]. Consistently, CCL3-CCR5 and CCL4-CCR5 interaction pairs were also detected between CD8 + effector T cells and various T cell subsets in our study. Interestingly, we noted a correlation between higher CCL4 expression in CD8 + effector T cells and a more favorable pathological response to NAT, even in the comparison between pCR and MPR groups (Fig. 3G). This finding aligns with a prior study demonstrating a link between elevated CCL4 expression, increased intra-tumoral CD8 + T cells, and prolonged overall survival in EC tumors, likely due to the capacity of CCL4 to attract CCR5 + cytolytic lymphocytes [81]. Besides, the presence of CCL4 in NK cells, along with its receptor SLC7A1 in cancer cells, and CCR8 in CD4 + naïve T cells/Treg cells signified an activation of NK cells [82], which may predict a more favorable pathological response in pCR patients before NAT.

Despite neoadjuvant anti-PD-1 combination therapy showing promising clinical benefits in reducing tumor cell contents, a substantial subset of patients exhibited minimal response to the treatment and were categorized as IPR patients in our study. We reported a series of immune checkpoint interaction pairs, including CTLA4-CD86, TIGIT-PVR, LGALS9-HAVCR2, and TNFSF4-TNFRSF4, which could offer further insights for the discovery and development of new therapeutic agents for ESCC.

While our study provided an unbiased and high-precision scRNA-seq analysis to decipher the TME in ESCC patients undergoing neoadjuvant chemo-immunotherapy, it is crucial to acknowledge the limitations of this pioneering study. One notable limitation was the restricted sample size, especially the scarcity of adequate paired samples for each patient before and after NAT, potentially limiting our ability to discern differences among various pathological response subgroups in ESCC patients. However, it is important to recognize the challenges associated with collecting paired samples for single-cell analysis, considering the relatively innovative nature of the treatment approach. Clinical constraints, such as the necessity for patients to receive the same treatment, the requirement for samples to meet sequencing criteria, and the technical demands of scRNA-seq necessitating fresh sample dissociation, all add complexity to the process. Nevertheless, it is imperative to address this limitation by expanding the sample size in future investigations. Furthermore, the absence of external validation through techniques such as immunohistochemistry or immunofluorescence, or comparative analysis with an external dataset involving patients undergoing the same treatment, might potentially impact the generalizability and overall validity of our findings. Therefore, our findings remain exploratory and should be approached with caution, considering the limited sample size and the extent of validation provided. Further exploration and validation of our results are warranted in future studies.

## Conclusions

In conclusion, our study provides a comprehensive single-cell atlas of ESCC based on large-scale scRNA-seq results. We thoroughly assessed the development of cancer cells and T cell transcriptome, revealing promising biomarkers associated with the pathological response to NAT. Our findings have significant implications for drug development and precision medication in ESCC patients.

### Supplementary Information

**Additional file 1.** All supplementary figures (Fig. S1-Fig. S5).

**Additional file 2. **scRNA-seq quality control data.

**Additional file 3:** **Table S1.** Clinical characteristics of patients grouped by pathological response.

**Additional file 4.** Cell count data.

**Additional file 5.** Differential expression analysis data in cancer cells.

**Additional file 6.** Differential expression analysis data in CD8+ effector T cells.

**Additional file 7.** Differential expression analysis data in NK cells.

**Additional file 8.** Differential expression analysis data in Treg cells.

**Additional file 9.** Raw data used for CellphoneDB analysis.

## Data Availability

The sequencing data generated and utilized in this study have been deposited in the Genome Sequence Archive (GSA) of the National Genomics Data Center (accession number: PRJCA016745; release date: October 25, 2024) [83] that is available at https://ngdc.cncb.ac.cn/bioproject/browse/PRJCA016745. The count matrix data have been deposited in OMIX, China National Center for Bioinformation/Beijing Institute of Genomics, Chinese Academy of Sciences (accession number: OMIX005710) [84] that are available at https://ngdc.cncb.ac.cn/omix/release/OMIX005710. Other data generated in this study are available in Additional files.

## Abbreviations

CNV: Copy number variation
CTLA-4: Cytotoxic T lymphocyte-associated antigen-4
DEG: Differentially expressed gene
EAC: Esophageal adenocarcinoma
ESCC: Esophageal squamous cell carcinoma
EASC: Esophageal adenosquamous carcinoma
EC: Esophageal cancer
GO: Gene ontology
GSEA: Gene set enrichment analysis
ICIs: Immune checkpoint inhibitors
IFNγ: Interferon-gamma
IPR: Incomplete pathological response
MPR: Major pathological response
NAT: Neoadjuvant chemo-immunotherapy
PD-1: Programmed death 1
PD-L1: Programmed death ligand 1
pCR: Pathological complete response
UMI: Unique molecular identifier
UMAP: Uniform manifold approximation and projection
VRTCs: Viable residual tumor cells
SCENIC: Single-cell regulatory network inference and clustering
TCGA: The Cancer Genome Atlas
TME: Tumor microenvironment

## References

[1] Uhlenhopp DJ, Then EO, Sunkara T, Gaduputi V (2020). Epidemiology of esophageal cancer: update in global trends, etiology and risk factors. Clin J Gastroenterol.

[2] Sung H, Ferlay J, Siegel RL, Laversanne M, Soerjomataram I, Jemal A, Bray F (2021). Global Cancer Statistics 2020: GLOBOCAN Estimates of Incidence and Mortality Worldwide for 36 Cancers in 185 Countries. CA Cancer J Clin.

[3] Napier KJ, Scheerer M, Misra S (2014). Esophageal cancer: A Review of epidemiology, pathogenesis, staging workup and treatment modalities. World J Gastrointest Oncol.

[4] van Hagen P, Hulshof MC, van Lanschot JJ, Steyerberg EW, van Berge HMI, Wijnhoven BP (2012). Preoperative chemoradiotherapy for esophageal or junctional cancer. N Engl J Med.

[5] Yang H, Liu H, Chen Y, Zhu C, Fang W, Yu Z (2018). Neoadjuvant Chemoradiotherapy Followed by Surgery Versus Surgery Alone for Locally Advanced Squamous Cell Carcinoma of the Esophagus (NEOCRTEC5010): A Phase III Multicenter, Randomized. Open-Label Clinical Trial J Clin Oncol.

[6] Zhang Z, Zhang H (2017). Impact of neoadjuvant chemotherapy and chemoradiotherapy on postoperative cardiopulmonary complications in patients with esophageal cancer. Dis Esophagus.

[7] Ohigashi Y, Sho M, Yamada Y, Tsurui Y, Hamada K, Ikeda N (2005). Clinical significance of programmed death-1 ligand-1 and programmed death-1 ligand-2 expression in human esophageal cancer. Clin Cancer Res.

[8] Shu CA, Gainor JF, Awad MM, Chiuzan C, Grigg CM, Pabani A (2020). Neoadjuvant atezolizumab and chemotherapy in patients with resectable non-small-cell lung cancer: an open-label, multicentre, single-arm, phase 2 trial. Lancet Oncol.

[9] Schmid P, Salgado R, Park YH, Munoz-Couselo E, Kim SB, Sohn J (2020). Pembrolizumab plus chemotherapy as neoadjuvant treatment of high-risk, early-stage triple-negative breast cancer: results from the phase 1b open-label, multicohort KEYNOTE-173 study. Ann Oncol.

[10] Sun JM, Shen L, Shan MA, Enzinger P, Adenis A, Doi T (2021). Pembrolizumab plus chemotherapy versus chemotherapy alone for first-line treatment of advanced oesophageal cancer (KEYNOTE-590): a randomised, placebo-controlled, phase 3 study. Lancet.

[11] Janjigian YY, Shitara K, Moehler M, Garrido M, Salman P, Shen L (2021). First-line nivolumab plus chemotherapy versus chemotherapy alone for advanced gastric, gastro-oesophageal junction, and oesophageal adenocarcinoma (CheckMate 649): a randomised, open-label, phase 3 trial. Lancet.

[12] Patel Sandip Pravin and Kurzrock Razelle (2015). PD-L1 Expression as a Predictive Biomarker in Cancer Immunotherapy. Mol Cancer Ther.

[13] Ito S, Okano S, Morita M, Saeki H, Tsutsumi S, Tsukihara H (2016). Expression of PD-L1 and HLA Class I in Esophageal Squamous Cell Carcinoma: Prognostic Factors for Patient Outcome. Ann Surg Oncol.

[14] Lim SH, Hong M, Ahn S, Choi YL, Kim KM, Oh D (2016). Changes in tumour expression of programmed death-ligand 1 after neoadjuvant concurrent chemoradiotherapy in patients with squamous oesophageal cancer. Eur J Cancer.

[15] Chen Miao-Fen, Chen Ping-Tsung, Chen Wen-Cheng, Lu Ming-Shian, Lin Paul-Yang, Lee Kuan-Der (2016). The role of PD-L1 in the radiation response and prognosis for esophageal squamous cell carcinoma related to IL-6 and T-cell immunosuppression. Oncotarget.

[16] Rong L, Liu Y, Hui Z, Zhao Z, Zhang Y, Wang B (2019). PD-L1 expression and its clinicopathological correlation in advanced esophageal squamous cell carcinoma in a Chinese population. Diagn Pathol.

[17] Guo Wei, Wang Pan, Li Ning, Shao Fei, Zhang Hao, Yang Zhenlin, Li Renda , Gao Yibo, He  Jie (2018). Prognostic value of PD-L1 in esophageal squamous cell carcinoma: a meta-analysis. Oncotarget.

[18] Chen Kaiyan, Cheng Guoping, Zhang Fanrong, Zhang Nan, Li Dan, Jin Jiaoyue, Wu Junzhou, Ying Lisha , Mao Weimin, Su Dan (2016). Prognostic significance of programmed death-1 and programmed death-ligand 1 expression in patients with esophageal squamous cell carcinoma. Oncotarget.

[19] McLane LM, Abdel-Hakeem MS, Wherry EJ (2019). CD8 T Cell Exhaustion During Chronic Viral Infection and Cancer. Annu Rev Immunol.

[20] Li C, Zhao S, Zheng Y, Han Y, Chen X, Cheng Z (2021). Preoperative pembrolizumab combined with chemoradiotherapy for oesophageal squamous cell carcinoma (PALACE-1). Eur J Cancer.

[21] van den Ende T, de Clercq NC, van Berge HMI, Gisbertz SS, Geijsen ED, Verhoeven RHA (2021). Neoadjuvant Chemoradiotherapy Combined with Atezolizumab for Resectable Esophageal Adenocarcinoma: A Single-arm Phase II Feasibility Trial (PERFECT). Clin Cancer Res.

[22] Yang W, Xing X, Yeung SJ, Wang S, Chen W, Bao Y (2022). Neoadjuvant programmed cell death 1 blockade combined with chemotherapy for resectable esophageal squamous cell carcinoma. J Immunother Cancer.

[23] He W, Leng X, Mao T, Luo X, Zhou L, Yan J (2022). Toripalimab Plus Paclitaxel and Carboplatin as Neoadjuvant Therapy in Locally Advanced Resectable Esophageal Squamous Cell Carcinoma. Oncologist.

[24] Carroll TM, Chadwick JA, Owen RP, White MJ, Kaplinsky  J, Peneva I (2023). Tumor monocyte content predicts immunochemotherapy outcomes in esophageal adenocarcinoma. Cancer Cell.

[25] Gocher AM, Workman CJ, Vignali DAA (2022). Interferon-gamma: teammate or opponent in the tumour microenvironment?. Nat Rev Immunol.

[26] von Locquenghien M, Rozalén C, Celià-Terrassa T. Interferons in cancer immunoediting: sculpting metastasis and immunotherapy response. J Clin Invest. 2021;131:e143296. https://www.ncbi.nlm.nih.gov/pmc/articles/PMC7773346/. 10.1172/JCI143296 PMC7773346 33393507

[27] Jorgovanovic D, Song M, Wang L, Zhang Y (2020). Roles of IFN-gamma in tumor progression and regression: a review. Biomark Res.

[28] Saleiro D, Platanias LC (2019). Interferon signaling in cancer. Non-canonical pathways and control of intracellular immune checkpoints. Semin Immunol.

[29] Mojic M, Takeda K, Hayakawa Y (2017). The Dark Side of IFN-γ: Its Role in Promoting Cancer Immunoevasion. Int J Mol Sci.

[30] Wen J, Fang S, Hu Y, Xi M, Weng Z, Pan C (2022). Impacts of neoadjuvant chemoradiotherapy on the immune landscape of esophageal squamous cell carcinoma. EBioMedicine.

[31] Croft W, Evans RPT, Pearce H, Elshafie M, Griffiths EA, Moss P (2022). The single cell transcriptional landscape of esophageal adenocarcinoma and its modulation by neoadjuvant chemotherapy. Mol Cancer.

[32] Daiko H, Kato K (2020). Updates in the 8th edition of the TNM staging system for esophagus and esophagogastric junction cancer. Jpn J Clin Oncol.

[33] Yan X, Duan H, Ni Y, Zhou Y, Wang X, Qi H (2022). Tislelizumab combined with chemotherapy as neoadjuvant therapy for surgically resectable esophageal cancer: A prospective, single-arm, phase II study (TD-NICE). Int J Surg.

[34] Dura B, Choi JY, Zhang K, Damsky W, Thakral D, Bosenberg M (2019). scFTD-seq: freeze-thaw lysis based, portable approach toward highly distributed single-cell 3' mRNA profiling. Nucleic Acids Res.

[35] Stuart T, Butler A, Hoffman P, Hafemeister C, Papalexi E, Mauck WM (2019). Comprehensive Integration of Single-Cell Data. Cell.

[36] Korsunsky I, Millard N, Fan J, Slowikowski K, Zhang F, Wei K (2019). Fast, sensitive and accurate integration of single-cell data with Harmony. Nat Methods.

[37] Becht E, McInnes L, Healy J, Dutertre CA, Kwok IW, Ng LG, et al. Dimensionality reduction for visualizing single-cell data using UMAP. Nat Biotechnol. 2019;37(1):38–44. 10.1038/nbt.4314 30531897

[38] Cortal A, Martignetti L, Six E, Rausell A (2021). Gene signature extraction and cell identity recognition at the single-cell level with Cell-ID. Nat Biotechnol.

[39] Sun H, Zhang L, Wang Z, Gu D, Zhu M, Cai Y (2023). Single-cell transcriptome analysis indicates fatty acid metabolism-mediated metastasis and immunosuppression in male breast cancer. Nat Commun.

[40] Wang J, Su M, Wei N, Yan H, Zhang J, Gong Y (2023). Chronic Active Epstein-Barr Virus Disease Originates from Infected Hematopoietic Stem Cells. Blood.

[41] Zhang L, Du F, Jin Q, Sun L, Wang B, Tan Z (2023). Identification and Characterization of CD8(+) CD27(+) CXCR3(-) T Cell Dysregulation and Progression-Associated Biomarkers in Systemic Lupus Erythematosus. Adv Sci (Weinh).

[42] Tickle Timothy, Georgescu Christophe, Tirosh Itay. Inferring CNV from Single-Cell RNA-Seq**.** 2018, Github. Available from: https://github.com/broadinstitute/inferCNV.

[43] Raivo Kolde, *Pretty heatmaps*. 2018, Github. Available from: https://github.com/raivokolde/pheatmap.

[44] Guo M, Bao EL, Wagner M, Whitsett JA, Xu Y (2017). SLICE: determining cell differentiation and lineage based on single cell entropy. Nucleic Acids Res.

[45] Qiu X, Hill A, Packer J, Lin D, Ma YA, Trapnell C (2017). Single-cell mRNA quantification and differential analysis with Census. Nat Methods.

[46] DeTomaso D, Yosef N (2021). Hotspot identifies informative gene modules across modalities of single-cell genomics. Cell Syst.

[47] DeTomaso D, Yosef N. Hotspot identifies informative gene modules across modalities of single-cell genomics. 2021. Github. Available from: https://github.com/YosefLab/Hotspot. 10.1016/j.cels.2021.04.005 33951459

[48] Van de Sande B, Flerin C, Davie K, De Waegeneer M, Hulselmans G, Aibar S (2020). A scalable SCENIC workflow for single-cell gene regulatory network analysis. Nat Protoc.

[49] Aibar S, Gonzalez-Blas CB, Moerman T, Huynh-Thu VA, Imrichova H, Hulselmans G (2017). SCENIC: single-cell regulatory network inference and clustering. Nat Methods.

[50] Yu G, Wang LG, Han Y, He QY (2012). clusterProfiler: an R package for comparing biological themes among gene clusters. OMICS.

[51] Efremova M, Vento-Tormo M, Teichmann SA, Vento-Tormo R (2020). Cell PhoneDB: inferring cell-cell communication from combined expression of multi-subunit ligand-receptor complexes. Nat Protoc.

[52] Sarah Teichmann, cellphonedb. 2020, Github. Available from: https://github.com/Teichlab/cellphonedb.

[53] Barbie DA, Tamayo P, Boehm JS, Kim SY, Moody SE, Dunn IF (2009). Systematic RNA interference reveals that oncogenic KRAS-driven cancers require TBK1. Nature.

[54] Peng J, Sun BF, Chen CY, Zhou JY, Chen YS, Chen H (2019). Single-cell RNA-seq highlights intra-tumoral heterogeneity and malignant progression in pancreatic ductal adenocarcinoma. Cell Res.

[55] Zhang M, Yang H, Wan L, Wang Z, Wang H, Ge C (2020). Single-cell transcriptomic architecture and intercellular crosstalk of human intrahepatic cholangiocarcinoma. J Hepatol.

[56] Chen Z, Huang Y, Hu Z, Zhao M, Bian Y, Chen Z (2021). Dissecting the single-cell transcriptome network in patients with esophageal squamous cell carcinoma receiving operative paclitaxel plus platinum chemotherapy. Oncogenesis.

[57] Chen Z, Zhao M, Liang J, Hu Z, Huang Y, Li M (2021). Dissecting the single-cell transcriptome network underlying esophagus non-malignant tissues and esophageal squamous cell carcinoma. EBioMedicine.

[58] Cheng S, Li Z, Gao R, Xing B, Gao Y, Yang Y (2021). A pan-cancer single-cell transcriptional atlas of tumor infiltrating myeloid cells. Cell.

[59] Yang L, Zhang X, Hou Q, Huang M, Zhang H, Jiang Z (2019). Single-cell RNA-seq of esophageal squamous cell carcinoma cell line with fractionated irradiation reveals radioresistant gene expression patterns. BMC Genomics.

[60] Yao J, Cui Q, Fan W, Ma Y, Chen Y, Liu T (2020). Single-cell transcriptomic analysis in a mouse model deciphers cell transition states in the multistep development of esophageal cancer. Nat Commun.

[61] Mirzaei S, Paskeh MDA, Entezari M, Mirmazloomi SR, Hassanpoor A, Aboutalebi M (2022). SOX2 function in cancers: Association with growth, invasion, stemness and therapy response. Biomed Pharmacother.

[62] Shen LY, Zhou T, Du YB, Shi Q, Chen KN (2019). Targeting HOX/PBX dimer formation as a potential therapeutic option in esophageal squamous cell carcinoma. Cancer Sci.

[63] Kojima T, Shah MA, Muro K, Francois E, Adenis A, Hsu CH (2020). Randomized Phase III KEYNOTE-181 Study of Pembrolizumab Versus Chemotherapy in Advanced Esophageal Cancer. J Clin Oncol.

[64] Deng T, Wang H, Yang C, Zuo M, Ji Z, Bai M (2022). Single cell sequencing revealed the mechanism of PD-1 resistance affected by the expression profile of peripheral blood immune cells in ESCC. Front Immunol.

[65] Qu J, Zhao Q, Yang L, Ping Y, Zhang K, Lei Q (2021). Identification and characterization of prognosis-related genes in the tumor microenvironment of esophageal squamous cell carcinoma. Int Immunopharmacol.

[66] Zhai JS, Song JG, Zhu CH, Wu K, Yao Y, Li N (2016). Expression of APPL1 is correlated with clinicopathologic characteristics and poor prognosis in patients with gastric cancer. Curr Oncol.

[67] Ljunggren HG, Malmberg KJ (2007). Prospects for the use of NK cells in immunotherapy of human cancer. Nat Rev Immunol.

[68] Cheng M, Chen Y, Xiao W, Sun R, Tian Z (2013). NK cell-based immunotherapy for malignant diseases. Cell Mol Immunol.

[69] Shimon S, Tomoyuki Y, Takashi N, Masahiro O (2008). Regulatory T Cells and Immune Tolerance. Cell.

[70] Tanaka A, Sakaguchi S (2017). Regulatory T cells in cancer immunotherapy. Cell Res.

[71] Tanigawa K, Tsukamoto S, Koma YI, Kitamura Y, Urakami S, Shimizu M (2022). S100A8/A9 Induced by Interaction with Macrophages in Esophageal Squamous Cell Carcinoma Promotes the Migration and Invasion of Cancer Cells via Akt and p38 MAPK Pathways. Am J Pathol.

[72] Feng Z, Qu J, Liu X, Liang J, Li Y, Jiang J (2021). Integrated bioinformatics analysis of differentially expressed genes and immune cell infiltration characteristics in Esophageal Squamous cell carcinoma. Sci Rep.

[73] Yao J, Duan L, Huang X, Liu J, Fan X, Xiao Z (2021). Development and Validation of a Prognostic Gene Signature Correlated With M2 Macrophage Infiltration in Esophageal Squamous Cell Carcinoma. Front Oncol.

[74] Aldinucci D, Borghese C, Casagrande N (2020). The CCL5/CCR5 Axis in Cancer Progression. Cancers (Basel).

[75] Zeng Z, Lan T, Wei Y, Wei X (2022). CCL5/CCR5 axis in human diseases and related treatments. Genes Dis.

[76] Ruiz de Galarreta M, Bresnahan E, Molina-Sanchez P, Lindblad  KE, Maier B, Sia D (2019). beta-Catenin Activation Promotes Immune Escape and Resistance to Anti-PD-1 Therapy in Hepatocellular Carcinoma. Cancer Discov.

[77] Huffman AP, Lin JH, Kim SI, Byrne KT, Vonderheide RH (2020). CCL5 mediates CD40-driven CD4+ T cell tumor infiltration and immunity. JCI Insight.

[78] Bottcher JP, Bonavita E, Chakravarty P, Blees H, Cabeza-Cabrerizo M, Sammicheli S (2018). NK Cells Stimulate Recruitment of cDC1 into the Tumor Microenvironment Promoting Cancer Immune Control. Cell.

[79] Dangaj D, Bruand M, Grimm AJ, Ronet C, Barras D, Duttagupta PA (2019). Cooperation between Constitutive and Inducible Chemokines Enables T Cell Engraftment and Immune Attack in Solid Tumors. Cancer Cell.

[80] Samson M, Labbe O, Mollereau C, Vassart G, Parmentier M (1996). Molecular cloning and functional expression of a new human CC-chemokine receptor gene. Biochemistry.

[81] Liu JY, Li F, Wang LP, Chen XF, Wang D, Cao L (2015). CTL- vs Treg lymphocyte-attracting chemokines, CCL4 and CCL20, are strong reciprocal predictive markers for survival of patients with oesophageal squamous cell carcinoma. Br J Cancer.

[82] Oliva A, Kinter AL, Vaccarezza M, Rubbert A, Catanzaro A, Moir S (1998). Natural killer cells from human immunodeficiency virus (HIV)-infected individuals are an important source of CC-chemokines and suppress HIV-1 entry and replication in vitro. J Clin Invest.

[83] Ji Gang, Yang Qi, Wang Song, Yan Xiaolong, Ou Qiuxiang, Gong Li, Zhao Jinbo, Zhou Yongan, Tian Feng, Lei Jie, Mu Xiaorong, Wang Jian, Wang Tao, Wang Xiaoping, Sun Jianyong, Zhang Jipeng, Jia Chenghui, Jiang Tao, Zhao Ming-gao, Lu Qiang, *Single-cell profiling revealed tumor heterogeneity and microenvironment in surgically resectable esophageal squamous cell carcinoma with neoadjuvant chemoimmunotherapy*. 2023, PRJCA016745, Genome Sequence Archive. Available from: https://ngdc.cncb.ac.cn/bioproject/browse/PRJCA016745.

[84] Ji Gang, Yang Qi, Wang Song, Yan Xiaolong, Ou Qiuxiang, Gong Li, Zhao Jinbo, Zhou Yongan, Tian Feng, Lei Jie, Mu Xiaorong, Wang Jian, Wang Tao, Wang Xiaoping, Sun Jianyong, Zhang Jipeng, Jia Chenghui, Jiang Tao, Zhao Ming-gao, Lu Qiang, *Single-cell profiling revealed tumor heterogeneity and microenvironment in surgically resectable esophageal squamous cell carcinoma with neoadjuvant chemoimmunotherapy*. 2024, OMIX005710, OMIX, China National Center for Bioinformation/Beijing Institute of Genomics, Chinese Academy of Sciences. Available from: https://ngdc.cncb.ac.cn/omix/release/OMIX005710.

