# Supplementary material for: Single-cell profiling of response to neoadjuvant chemo-immunotherapy in surgically resectable esophageal squamous cell carcinoma
Source: Genome Med. 2024 Apr 2;16:49. doi: 10.1186/s13073-024-01320-9 (PMC10985969; doi:10.1186/s13073-024-01320-9)
Supplement: Supplementary file 1 — Additional file 1. All supplementary figures (Fig. S1-Fig. S5). [file 13073_2024_1320_MOESM1_ESM.docx]

**
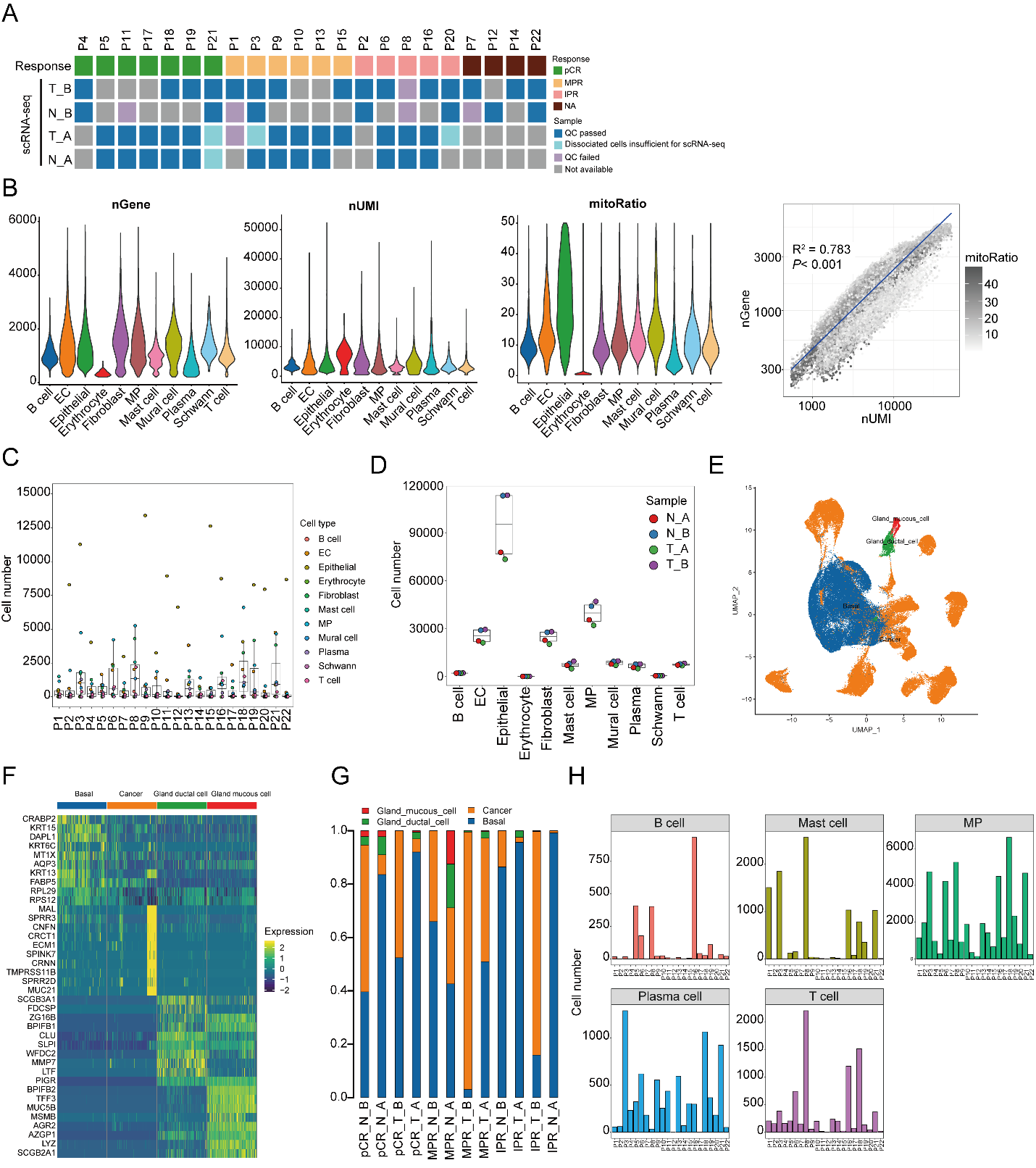
**

### **Fig. S1. An overview of single-cell sequencing information.**

(**A**) Sample availability for each patient. The pathological response of each patient to neoadjuvant anti-PD-1 combination therapy was pathologically examined and categorized into three categories: pathological complete response (pCR, green), major pathological response (MPR, orange), and incomplete pathological response (IPR, pink). The pathological response status of four patients (maroon) was unknown due to surgery cancellations. Samples with insufficient cells after single-cell dissociation (turquoise) were excluded from scRNA-seq. Only samples that passed the quality control (QC, steel blue) were subjected to subgroup analysis to determine the relationship between single-cell transcriptional profiles and pathological response. (**B**) Diagrams showing the quality control data of scRNA-seq analysis, including the number of genes per cell (nGene), the number of UMIs per cell (nUMI), the mitochondrial ratio (mitoRatio), and the interplay between these three metrics. (**C, D**) Box plots show the number of each cell cluster by the patient (C) and sample (D), respectively. (**E**) UMAP embedding of epithelial cells overlaid with clustered cell type annotations. (**F**) Average expression profile of canonical marker genes to separate epithelial cell subtypes. (**G**) The proportion of epithelial cell subsets in 12 patient subgroups. (**H**) Bar plots illustrating the cell number count of immune cells, including B cells, mast cells, MP cells, plasma cells, and T cells, across all 22 patients.


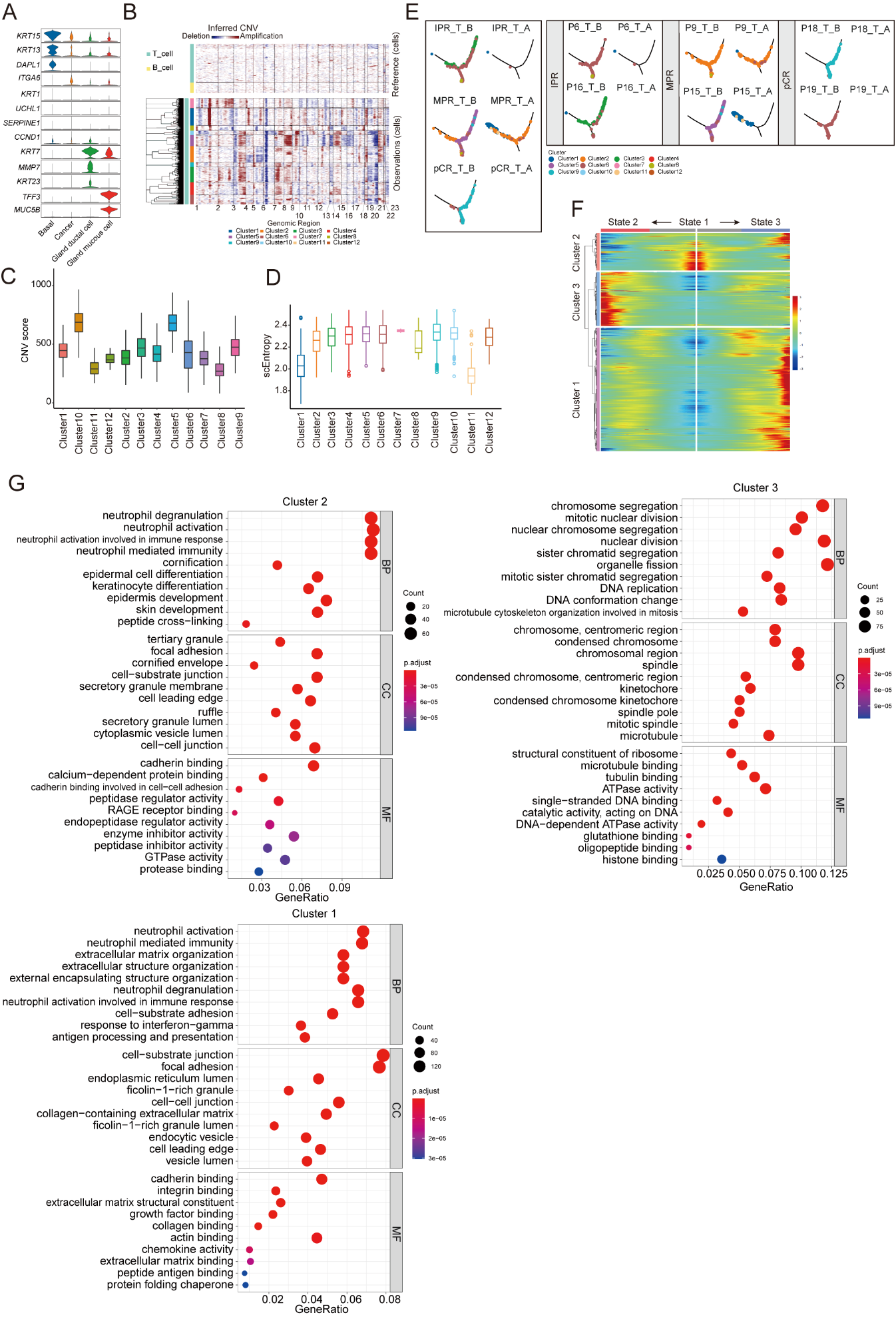


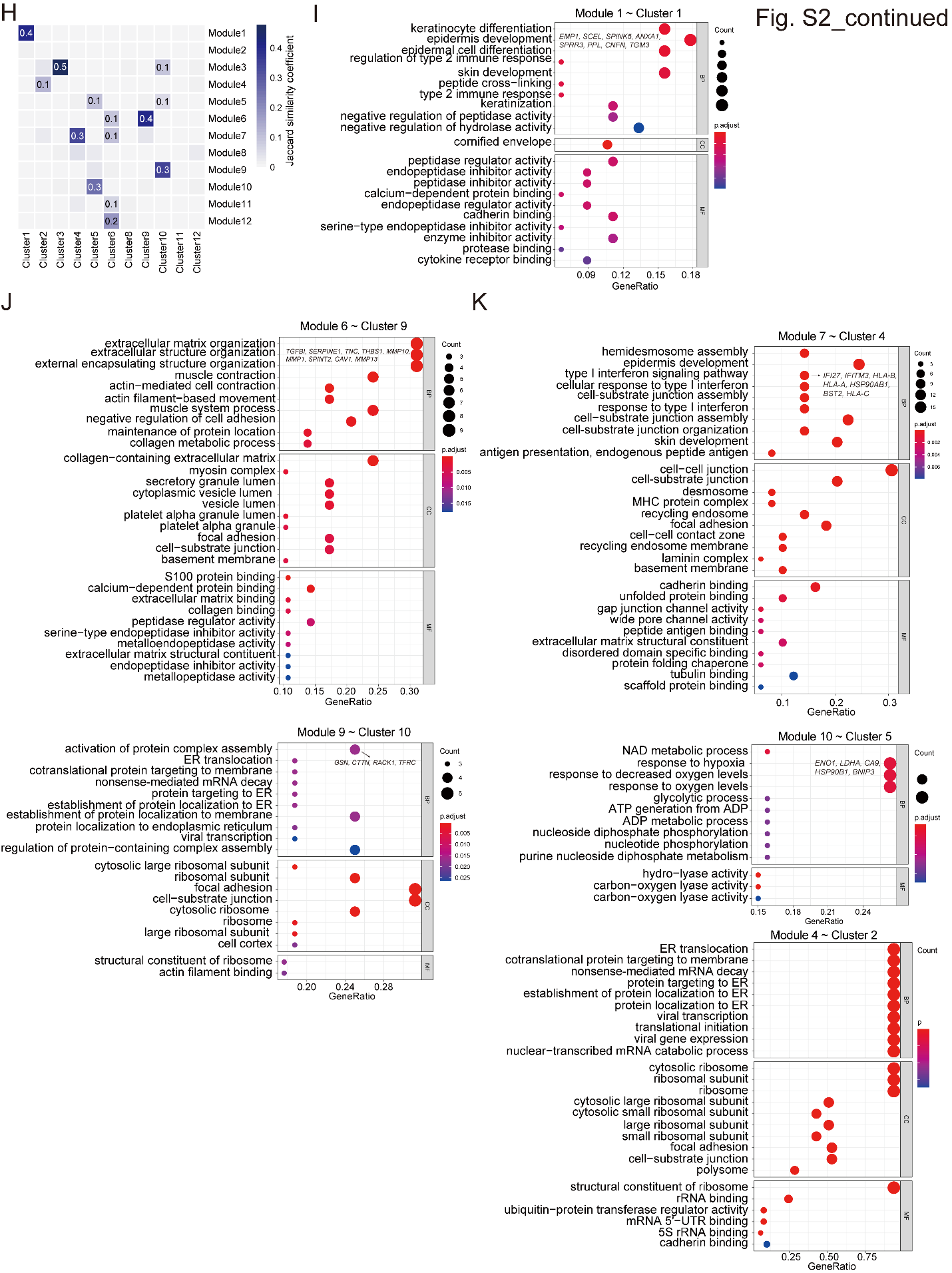


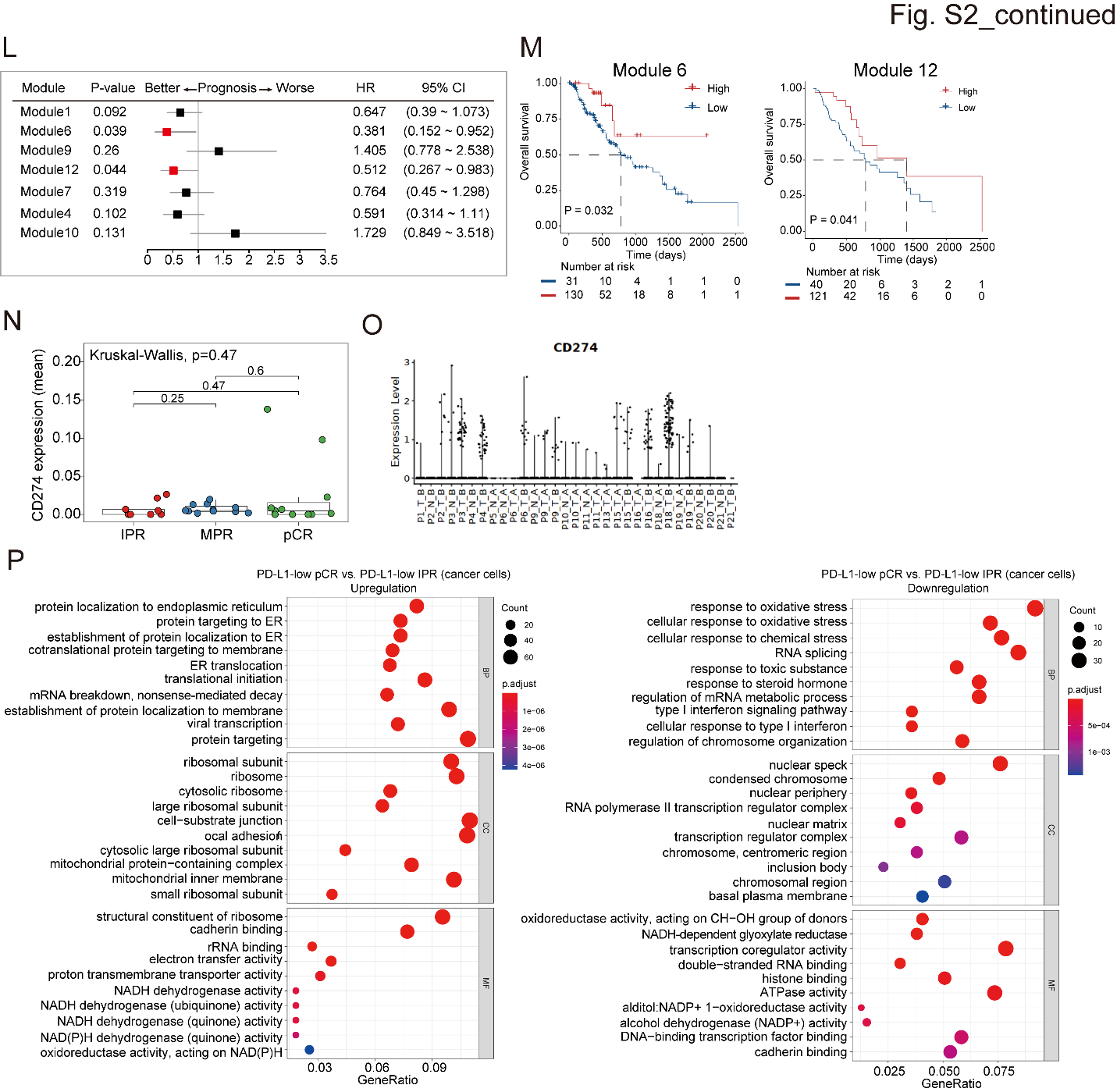


### **Fig. S2. Cancer cell differentiation and its association with pathological response to neoadjuvant chemo-immunotherapy.**

(**A**) Violin plots illustrating the expression of canonical marker genes in epithelial cell subsets. (**B**) Heatmap shows the average expression of copy number variations (CNVs) across chromosomal intervals, using T cells and B cells as references. (**C**) The box plots show the CNV scores of the 12 cancer clusters classified by unsupervised clustering analysis. (**D**) The box plots show the single-cell entropy (scEntropy) across the 12 cancer cell clusters. (**E**) Faceted pseudotime trajectories indicate the distribution of each cancer cell cluster in paired groups and individual patients. (**F**) Dynamic changes in gene expression of cancer cells during the transition. (**G**) Gene Ontology (GO) enrichment analysis of gene clusters during the transition. The X-axis represents the ratio of mRNAs enriched in GO terms. The Y-axis represents the enriched pathway. The color and size of each bubble represent enrichment significance and the number of related mRNAs enriched in the pathway, respectively. (**H**) Heatmap shows the Jaccard similarity coefficients between cancer cell clusters identified by unsupervised clustering analysis and gene modules identified by the hotspot analysis. (**I**) GO pathway enrichment analysis of module 1 showed a significant correlation with cancer 1 based on similarity coefficients. (**J**) GO pathway enrichment analysis of modules 6 and 9 that correspond to clusters 9 and 10 showing great response to neoadjuvant therapy of pCR patients. (**K**) GO pathway enrichment analysis of gene modules that correlated with cancer clusters with significant clinical response in MPR patients. (**L**) The forest plot demonstrates the relationship between each cancer cell-associated gene module and the overall survival of TCGA-ESCA patients based on the Cox regression model. HR, hazard ratio; CI, confidence interval. (**M**) Kaplan-Meier curves showing the overall survival of TCGA-ESCA patients with high or low expression of module 6 or module 12 genes. (**N, O**) Expression of *CD274* (*PD-L1*) in cancer cells of three patient cohorts (N) and each sample (O). (**P**) GO pathway enrichment analysis of cancer cells for baseline tumor of PD-L1-low pCR versus PD-L1-low IPR patients.


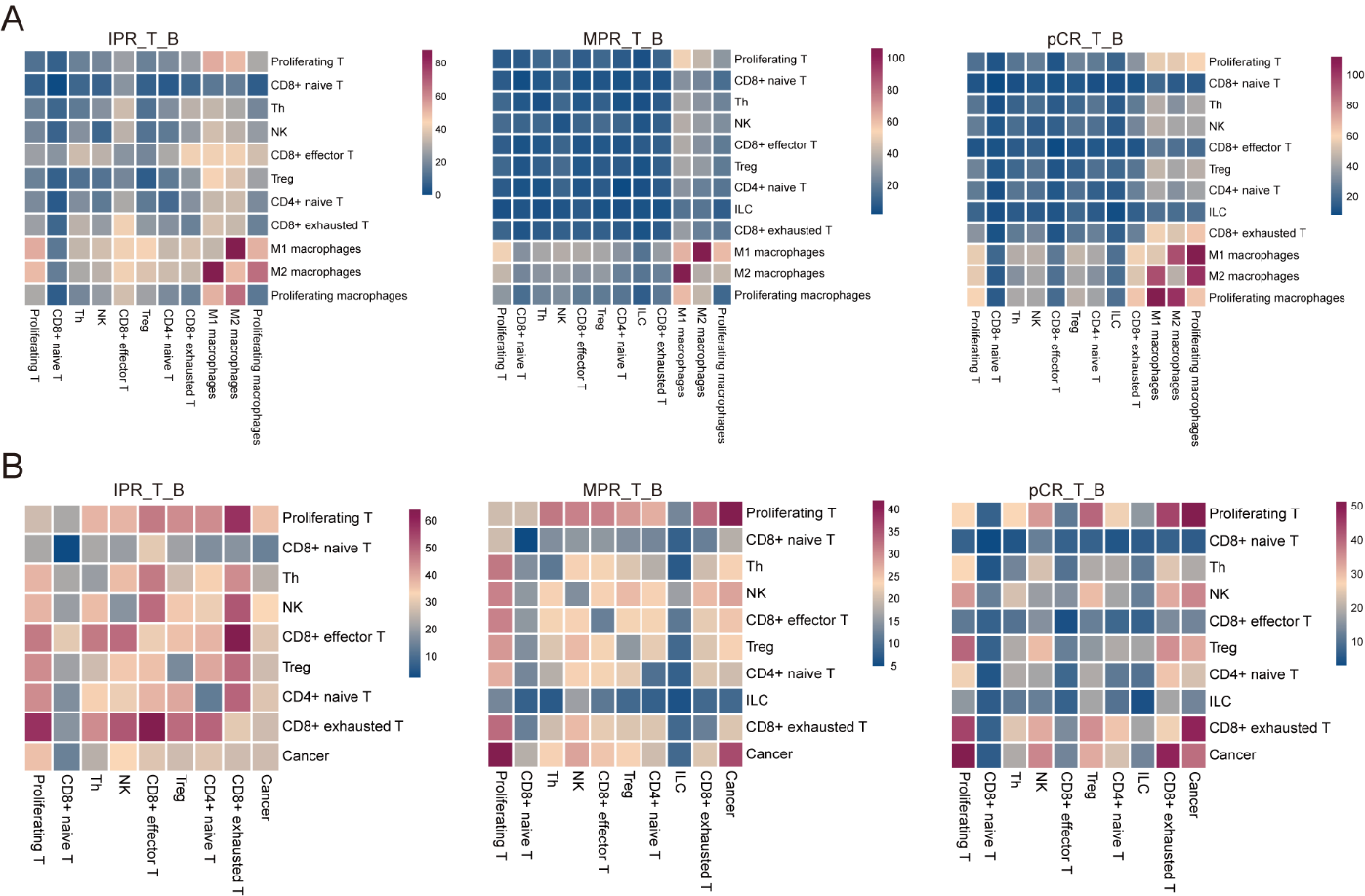


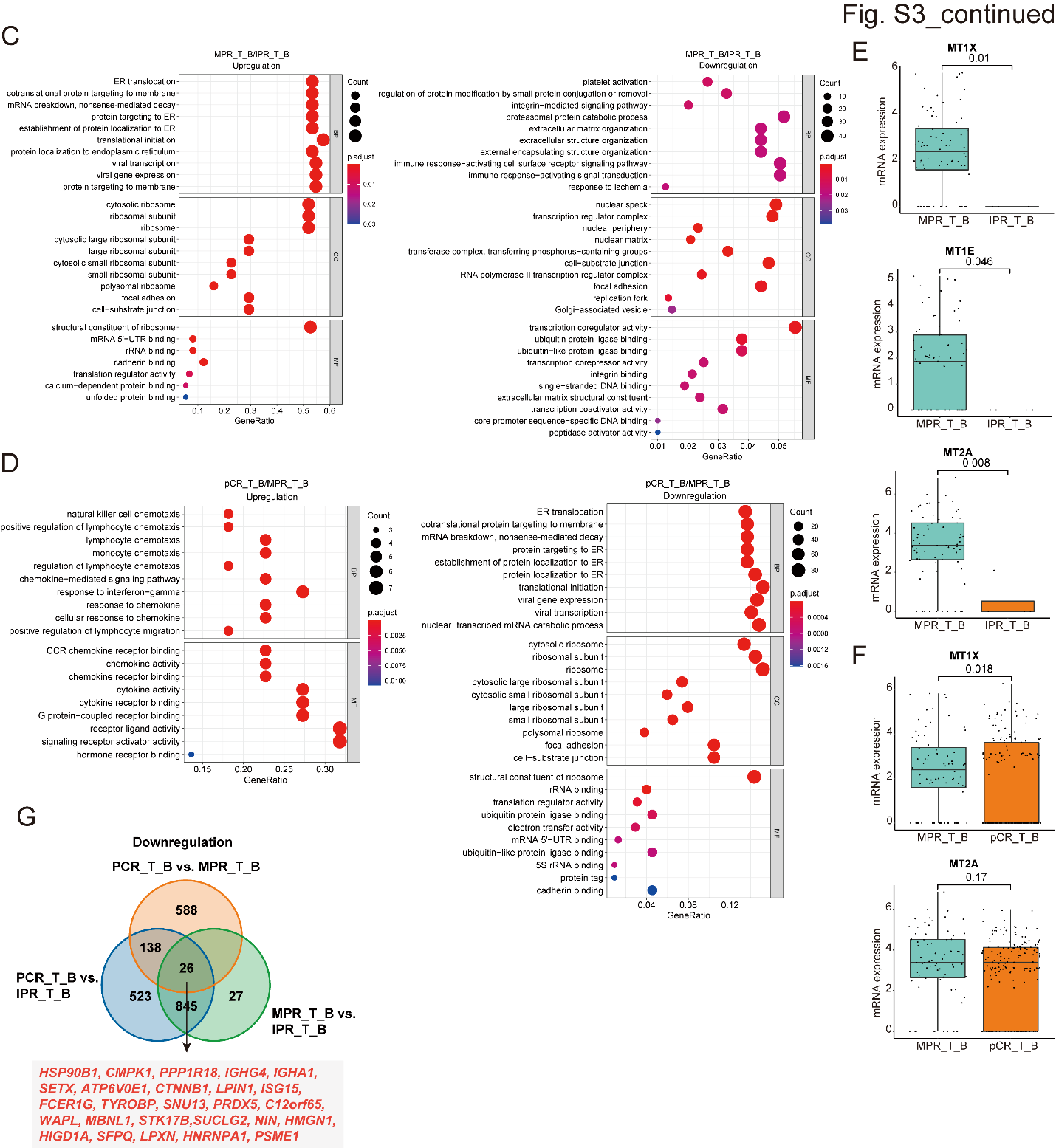


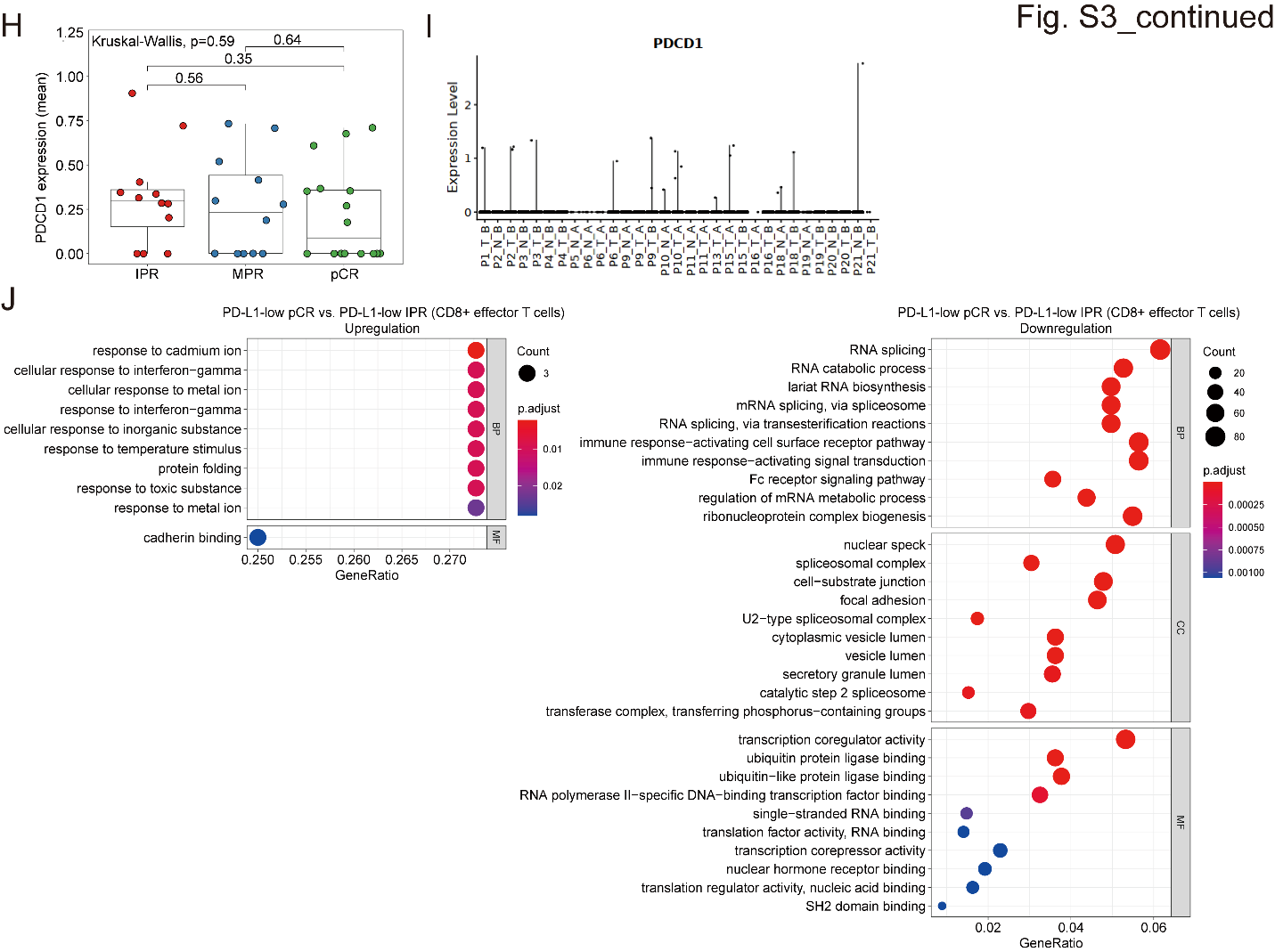


### **Fig. S3. Modulation of CD8+ effector T cells in the ESCC TME of patients with different pathological responses to NAT.**

(**A**) CellPhoneDB-generated heatmap shows the count of T cell-MP cell interactions in pre-treatment tumor samples of IPR, MPR, and pCR patients. (**B**) Heatmap shows T cell-T cell interactions in pre-treatment ESCC tumors of patients with different pathological responses to neoadjuvant chemo-immunotherapy. (**C**) Gene Ontology (GO) pathway enrichment analysis of CD8+ effector T cells obtained from MPR versus IPR patients. (**D**) GO pathway enrichment analysis of CD8+ effector T cells obtained from pCR versus MPR patients. (**E**) Expression of metallothionein family genes, including *MT1X*, *MT1E*, and *MT2A*, in MPR and IPR patients. (**F**) *MT1X* but not *MT2A* showed a significant down-regulation in pCR patients versus MPR patients. (**G**) The Venn diagram shows the intersections of three gene sets. Gene sets included down-regulated genes in CD8+ effector T cells obtained from pre-treatment tumors of patients with different pathological responses to treatment. (**H, I**) Expression of *PDCD1* (*PD-1*) in T cells of three patient cohorts (H) and each sample (I). (**J**) GO pathway enrichment analysis of CD8+ effector T cells for baseline tumor of PD-L1-low pCR versus PD-L1-low IPR patients.


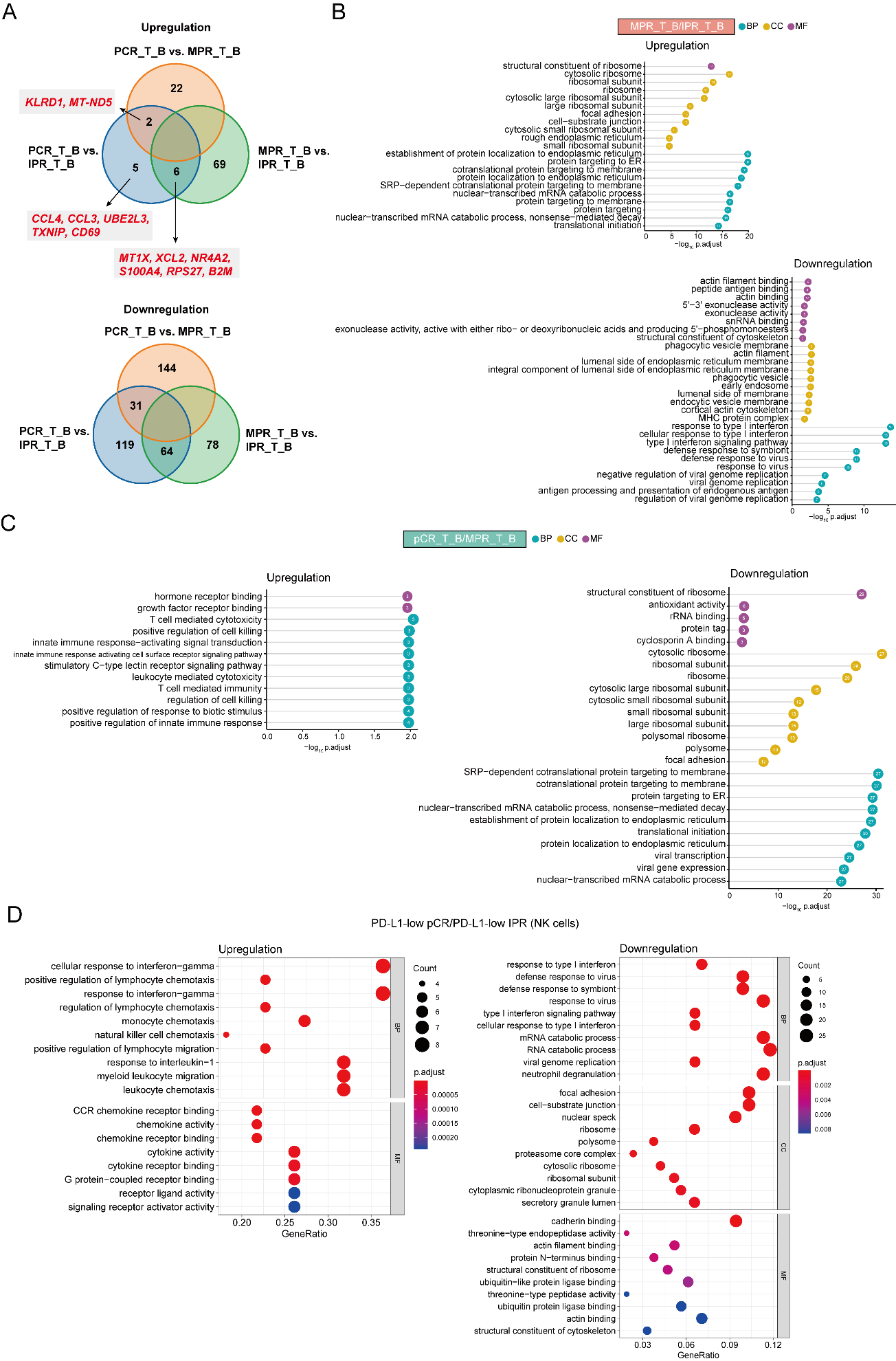


### **Fig. S4. Supplementary pathway enrichment analysis of NK cells.**

(**A**) The Venn diagrams show the intersections among three sets of genes comparing upregulated (top) and downregulated (bottom) genes in the DEG analysis of Natural killer (NK) cells. Gene sets were based on baseline tumors of patients who exhibit varying pathological responses to neoadjuvant chemo-immunotherapy. (**B**) Gene Ontology (GO) pathway enrichment analysis of NK cells obtained from MPR versus IPR patients. (**C**) GO pathway enrichment analysis of NK cells obtained from pCR versus MPR patients. (**D**) GO pathway enrichment analysis of NK cells for baseline tumor of PD-L1-low pCR versus PD-L1-low IPR patients.


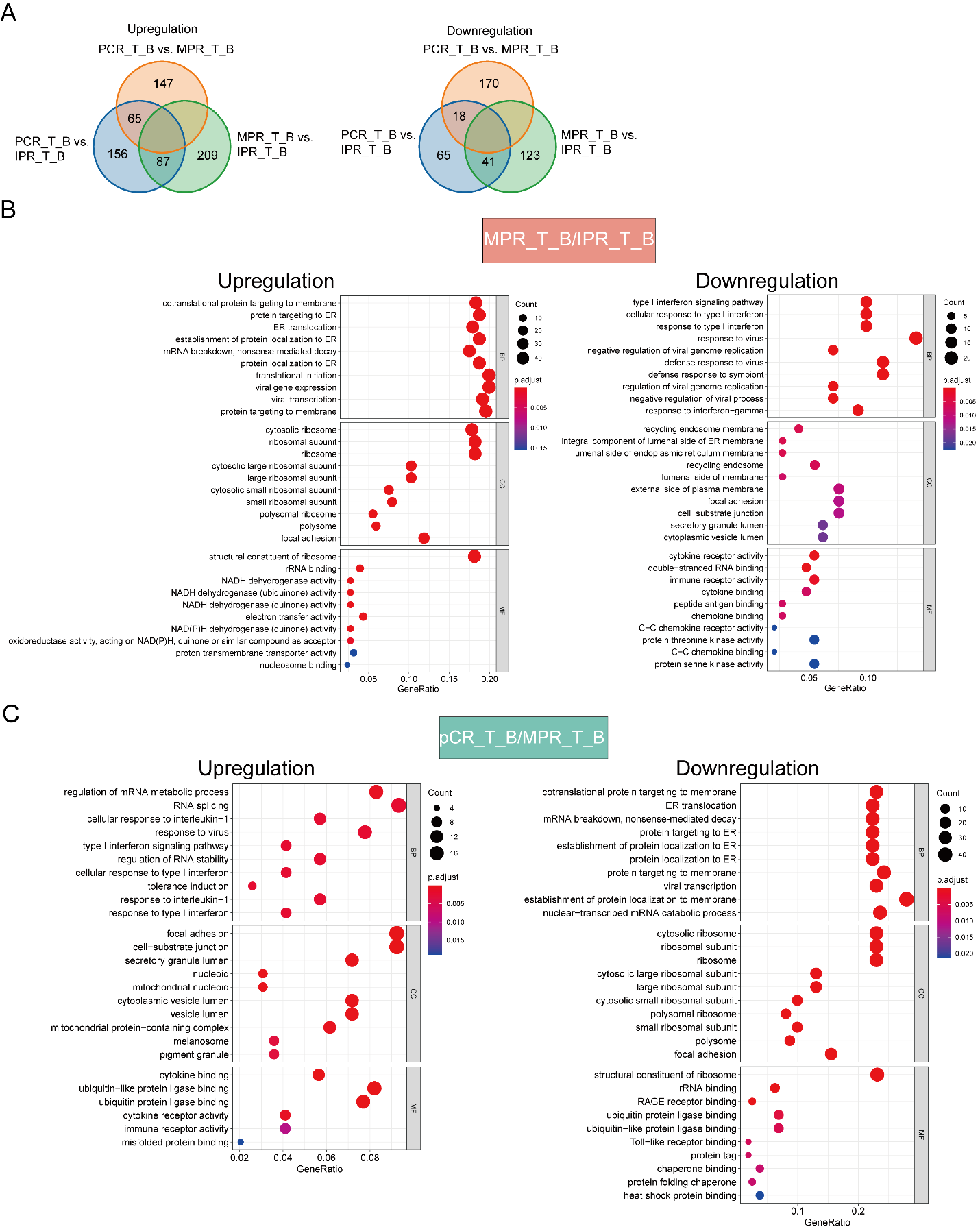


### **Fig. S5. Supplementary pathway enrichment analysis of Treg cells.**

(**A**) The Venn diagrams show the intersections among three sets of genes comparing upregulated (top) and downregulated (bottom) genes in the DEG analysis of regulatory T (Treg) cells. Gene sets were based on baseline tumors of patients who exhibit varying pathological responses to neoadjuvant chemo-immunotherapy. (**B**) GO pathway enrichment analysis of Treg cells obtained from MPR versus IPR patients. (**C**) GO pathway enrichment analysis of Treg cells obtained from pCR versus MPR patients.
