# Supplementary material for: Single-cell profiling of response to neoadjuvant chemo-immunotherapy in surgically resectable esophageal squamous cell carcinoma
Source: Genome Med. 2024 Apr 2;16:49. doi: 10.1186/s13073-024-01320-9 (PMC10985969; doi:10.1186/s13073-024-01320-9)
Supplement: Supplementary file 3 — Additional file 3: Table S1. Clinical characteristics of patients grouped by pathological response. [file 13073_2024_1320_MOESM3_ESM.docx]

| **Table S1. Clinical characteristics of patients with a definitive pathological response (N=18)** | | | | |
| --- | --- | --- | --- | --- |
| Characteristic | N (%) | | | *P* value* |
|  | IPR (N=5) | MPR (N=6) | pCR (N=7) |  |
| Sex |  |  |  | 0.092 |
| Female | 0 | 4 (66.7) | 3 (42.9) |  |
| Male | 5 (100) | 2 (33.3) | 4 (57.1) |  |
| Age |  |  |  | 0.424 |
| >65 | 3 (60.0) | 2 (33.3) | 5 (71.4) |  |
| ≤65 | 2 (40.0) | 4 (66.7) | 2 (28.6) |  |
| Smoking history |  |  |  | 0.02 |
| Never smokers | 1 (20.0) | 6 (100) | 5 (71.4) |  |
| Former/current smokers | 4 (80.0) | 0 | 2 (28.6) |  |
| Clinical stage |  |  |  | 0.84 |
| II | 3 (60.0) | 2 (33.3) | 3 (42.9) |  |
| III | 2 (40.0) | 4 (66.7) | 4 (57.1) |  |
| Histological subtype |  |  |  | 0.278 |
| EASC | 1 (20.0) | 0 | 0 |  |
| ESCC | 4 (80.0) | 6 (100) | 7 (100) |  |
| TPS |  |  |  | 0.009 |
| <1% | 5 (100) | 6 (100) | 2 (28.6) |  |
| 1%≤TPS<50% | 0 | 0 | 1 (14.3) |  |
| ≥50% | 0 | 0 | 4 (57.1) |  |
| CPS |  |  |  | 0.198 |
| <1 | 3 (60.0) | 3 (50.0) | 2 (28.6) |  |
| 1≤TPS<10 | 1 (20.0) | 2 (33.3) | 0 |  |
| 10≤TPS<50 | 1 (20.0) | 1 (16.7) | 1 (14.3) |  |
| ≥50 | 0 | 0 | 4 (57.1) |  |
| **P* values were calculated based on Fisher’s exact test. IPR, incomplete pathological response; MPR, major pathological response; pCR, pathological complete response; ESCC, esophageal squamous cell carcinoma; EASC, esophageal adenosquamous carcinoma; TPS, tumor proportion score; CPS, combined positive score | | | | |
